# Modulation of Symbiotic Compatibility by Rhizobial Zinc Starvation Machinery

**DOI:** 10.1128/mBio.03193-19

**Published:** 2020-02-18

**Authors:** Pan Zhang, Biliang Zhang, Jian Jiao, Shi-Qi Dai, Wen-Xin Chen, Chang-Fu Tian

**Affiliations:** a State Key Laboratory of Agrobiotechnology, MOA Key Laboratory of Soil Microbiology, Rhizobium Research Center, College of Biological Sciences, China Agricultural University, Beijing, China; University of California, Berkeley

**Keywords:** *Sinorhizobium*, soybean, zinc, nodulation, compatibility

## Abstract

Pathogenic bacteria need high-affinity zinc uptake systems to counteract the nutritional immunity exerted by infected hosts. However, our understanding of zinc homeostasis in mutualistic systems such as the rhizobium-legume symbiosis is limited. Here, we show that the conserved high-affinity zinc transporter ZnuABC and accessory transporter proteins (Zip1, Zip2, and c06450) made cumulative contributions to nodulation of the broad-host-range strain Sinorhizobium fredii CCBAU45436. Zur acted as a zinc-dependent repressor for the *znuC-znuB-zur* operon, *znuA*, and *c06450* by binding to the associated Zur box, but did not regulate *zip1* and *zip2*. ZnuABC was the major zinc transporter. Combined mutants lacking *znuA* and one of the three accessory genes had more severe defects in nodulation and growth under zinc starvation conditions than the *znuA* mutant, though rhizoplane colonization by these mutants was not impaired. In contrast to the elite strain CCBAU45436, more drastic symbiotic defects were observed for the *znuA* mutants of other *Sinorhizobium* strains, which lack at least one of the three accessory genes in their genomes and are characterized by their limited host range and geographical distribution. The *znu*-derived mutants showed a higher expression level of *nod* genes involved in Nod factor biosynthesis and a reduced expression of genes encoding a type three secretion system and its effector NopP, which can interfere with the host immune system. Application of exogenous zinc restored the nodulation ability of these *znu*-derived mutants. Therefore, the conserved ZnuABC and accessory components in the zinc starvation machinery play an important role in modulating symbiotic compatibility.

## Abstract (precis)

The rhizobium-legume symbiosis contributes around 65% of biological nitrogen fixation in agriculture systems and is critical for sustainable agriculture by reducing the amount of chemical nitrogen fertilizer being used. Rhizobial inocula have been commercialized for more than 100 years, but the efficiency of inoculation can vary among legume cultivars, field sites, and years. These long-lasting challenging problems impede the establishment of a sustainable agriculture, particularly in developing countries. Here, we report that rhizobial zinc starvation machinery containing a conserved high-affinity zinc transporter and accessory components makes cumulative contributions to modulating rhizobial symbiotic compatibility. This work highlights a critical role of largely unexplored nutritional immunity in the rhizobium-legume symbiosis, which makes zinc starvation machinery an attractive target for improving rhizobial symbiotic compatibility.

## INTRODUCTION

Zinc (Zn) is an essential trace metal for all living organisms since it functions as a catalytic cofactor or structural element in a large number of proteins (1). In bacteria, zinc homeostasis is tightly maintained for optimal growth and survival by coordinating different uptake, storage and export systems, which are regulated by separate regulator proteins (2–4). Zinc sufficiency, in most bacteria that have been investigated, is sensed by Zur (zinc uptake regulator), belonging to the well-known Fur (iron-uptake regulator) family, which acts as a repressor for zinc uptake genes (3, 5–7). However, this regulation can be complex due to the variation in the number and characteristics of zinc uptake systems in different genomic backgrounds. In Escherichia coli, the low-affinity zinc importer ZupT, in the ZIP (zinc-iron permease) family, exerted a cumulative effect with the conserved high-affinity zinc importer ZnuABC on bacterial fitness during urinary tract infection, though the loss of ZupT had a less significant effect on zinc uptake than *znuABC* disruption (8). A similar conclusion was drawn for ZupT of Salmonella enterica (9). In contrast to the less-studied low-affinity zinc transporter, the conserved ZnuABC has been widely studied in many bacteria, such as E. coli, S. enterica, Bacillus subtilis, Yersinia pestis, Yersinia ruckeri, Streptomyces coelicolor, and Agrobacterium tumefaciens (6, 10–16). It has been established that ZnuABC plays an essential role in bacterial growth under zinc-deplete conditions and dramatically affects virulence of diverse bacteria (14, 17–19). However, a few exceptions exist. In Y. pestis, disruption of *znuABC* has no impact on virulence, though ZnuABC is the major zinc importer (20). In A. tumefaciens, TroCBA rather than ZnuABC predominately functions for zinc uptake (6). ZinT can participate in zinc uptake as an auxiliary component of ZnuA in E. coli and S. enterica (16, 17, 21), whereas in A. tumefaciens, ZinT may interact with the dominant high-affinity zinc uptake transporter TroCBA rather than with ZnuA (6, 15).

In contrast to the intensive study of pathogenic bacteria, the role of zinc homeostasis in mutualistic interactions remains largely unexplored. The rhizobium-legume symbiosis is a model system of mutualistic interactions between bacteria and eukaryotes and has global impacts on the nitrogen cycle due to its ability to reduce atmospheric nitrogen into ammonium. Rhizobia need key symbiosis genes (*nod*, *nif*, and *fix*) to form nitrogen-fixing nodules on legumes, though diverse core and accessory functions are also required for optimizing the compatibility and efficiency of rhizobial interactions with legumes (22–26). It was recently reported that *znuABC* genes of a broad-host-range strain Sinorhizobium fredii CCBAU45436 were upregulated in nodules of cultivated soybean (Glycine max) and wild soybean (Glycine soja) compared to those in free-living rhizobial cells in rich medium (26, 27), implying that the nodule is a zinc-limiting condition for rhizobia. A deletion mutant of *znuA* in *S. fredii* CCBAU45436 formed ineffective nodules on *G. max* cv. JD17 and cv. C08 and induced a reduced number of effective nodules on *G. soja* accession W05 (26, 27). On the other hand, it was shown that a soybean zinc transporter GmZIP1 is localized on the membrane of the symbiosome, which harbors nitrogen-fixing rhizobia (called bacteroids) within nodule cells, and isolated soybean symbiosomes can take up zinc from the medium (28). The downregulation of a zinc transporter MtZIP6 in Medicago truncatula resulted in reductions of nitrogenase activity, the number of red nodules, and plant biomass (29). These findings imply a role of zinc uptake in rhizobial interactions with legumes at both nodulation and nitrogen-fixation stages. However, the host-dependent variation in the symbiotic performance of the *znuA* mutant of *S. fredii* CCBAU45436 (26, 27) suggests that unidentified low-affinity zinc uptake systems might be involved in rhizobial adaptations to different hosts. Moreover, it remains unknown how Znu and other potential zinc uptake systems are regulated in rhizobia and how rhizobia sense the fluctuating zinc levels.

To strengthen our knowledge on the role of zinc homeostasis in the rhizobium-legume symbiosis, we performed a systematic investigation of zinc-responding genes, particularly those zinc uptake genes within the genome of *S. fredii* CCBAU45436. First, *in vivo* transcriptional and translational analyses were used to test whether the conserved Zur works as a zinc-responding transcriptional factor. Then, the Zur regulon and zinc-responding metal transporters were identified by using transcriptome sequencing (RNA-seq) and reverse transcription-quantitative PCR (qRT-PCR) studies of the wild-type CCBAU45436 and the *zur* mutant under zinc-replete and -deplete conditions. Bioinformatics and *in vitro* gel shift assays using purified Zur protein were performed to identify direct targets of Zur. The role of Zur, ZnuABC, and other putative transporters in zinc uptake and symbiotic interactions were further investigated using individual or combined mutants of these genes. The symbiotic defects associated with *znu*-derived mutants can be overcome by adding replete zinc into the legume rhizosphere, and the underlying mechanism was also investigated. Findings obtained in this study highlighted the importance of the cumulative contributions by ZnuABC and accessory zinc uptake systems to modulating rhizobial nodulation signaling pathways.

## RESULTS

### Response of Zur to fluctuating zinc levels.

**FIG 1:**
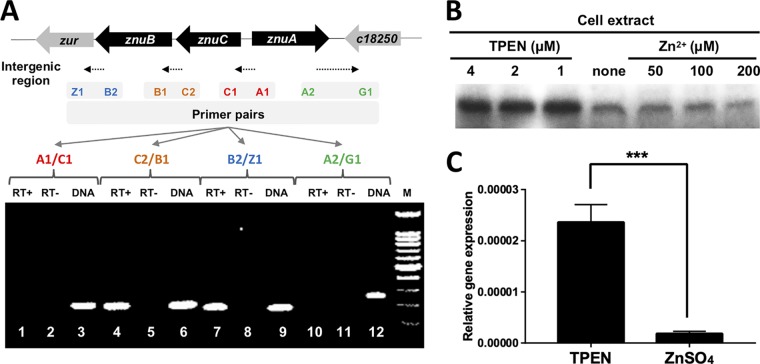
Expression of Zur under zinc excess and depleted conditions. (A) Cotranscription of *zur* with *znuC* and *znuB*. Transcription of four intergenic regions was tested by RT-PCR on total RNA isolated from wild-type CCBAU45436 in the M9 medium using the indicated primer pairs (see Table S2 in the supplemental material). Reverse transcriptase was added to the reaction in lanes 1, 4, 7, and 10 (RT+) but omitted from reactions in lanes 2, 5, 8, and 11 (RT−) to rule out the possibility of contamination by genomic DNA. Genomic DNA was amplified as a positive control in lanes 3, 6, 9, and 12 (DNA). M, 100 bp marker. (B) Western blot analysis of Zur. Exponentially grown CCBAU45436_3×Myc cells were either untreated or treated with various concentrations of chelator TPEN (4, 2, and 1 μM) or ZnSO_4_ (200, 100, and 50 μM) for 12 h before cell harvest. (C) qRT-PCR analysis of *zur* gene of wild-type CCBAU45436 in M9 medium supplemented with 200 μM ZnSO_4_ or 4 μM TPEN. ***, *P* < 0.001 by *t* test. Values are given as means ± standard deviations (SDs) from biological triplicates in three independent experiments.

A putative zinc uptake regulator gene *zur* (*c18210*) was found downstream from *znuC* and *znuB* (Fig. 1A). Reverse transcription PCR analysis showed that PCR products were obtained for fragments covering the intergenic regions between *znuB* and *zur* and between *znuC* and *znuB* but not for the regions between *znuA* and *znuC* and between *c18250* and *znuA* (Fig. 1A). Therefore, *znuC*, *znuB*, and *zur* constitute an operon.

c18210 is very similar (91.6% identity) to Zur in the model rhizobium Sinorhizobium meliloti 1021, and five conserved residues (H77, C87, C90, C128, and C130) critical for the specific binding to zinc ions are present in c18210 and Zur of other bacteria such as E. coli and B. subtilis (see Fig. S1 in the supplemental material). The protein level of Zur in CCBAU45436 was significantly elevated in medium supplemented with the zinc chelator TPEN [*N*,*N*,*N*′,*N*′-tetrakis(2-pyridinylmethyl)-1,2-ethanediamine)] compared to that with ZnSO_4_ or no treatment (Fig. 1B). The transcription level of *zur* was increased around 10-fold in the medium containing 4 μM TPEN compared to that in the medium with 200 μM ZnSO_4_ (*P* < 0.001) (Fig. 1C). These observations demonstrate that Zur expression is controlled by zinc levels.

10.1128/mBio.03193-19.1 FIG S1 Sequence alignment of c18210 with other bacterial Zur proteins. The protein sequence of SF45436_c18210 was aligned with Zur proteins from the following bacteria: Escherichia coli, Salmonella enterica, Klebsiella pneumoniae, Yersinia pestis, Vibrio cholerae, Pseudomonas aeruginosa, Xanthomonas campestris, Bacillus subtilis, ans Staphylococcus aureus using ClustalW. Five conserved residues (H77, C87, C90, C128, and C130) that are critical for the specific binding to zinc ions are indicated with triangles. Download FIG S1, PDF file, 0.1 MB. Copyright © 2020 Zhang et al. 2020 Zhang et al. This content is distributed under the terms of the Creative Commons Attribution 4.0 International license.

### Identification of Zur-regulated transporter genes responding to zinc depletion.

**FIG 2:**
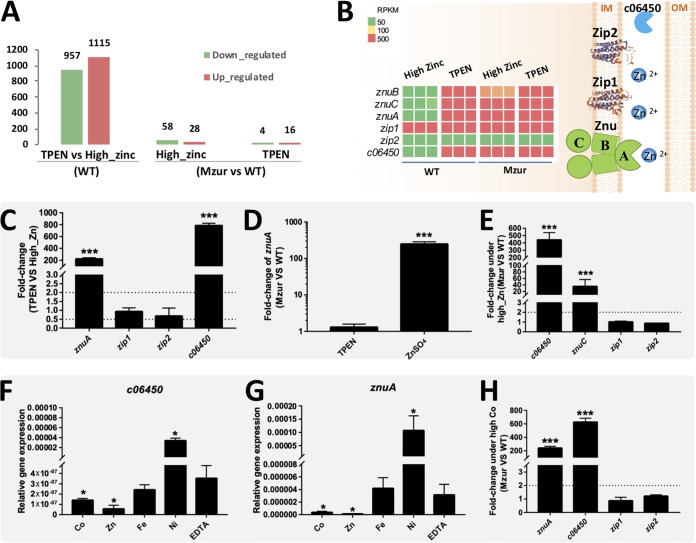
RNA-seq and qRT-PCR analyses of the Zur regulon of *S. fredii*. (A) RNA-seq analysis of transcriptomes of the wild-type CCBAU45436 (WT) and Mzur under low-zinc (4 μM TPEN) and high-zinc (200 μM ZnSO_4_) conditions (see Table S1A). The numbers of down- and upregulated genes are shown for WT under low-zinc conditions compared with those under high-zinc conditions (left), and for Mzur compared with WT under either high-zinc or low-zinc conditions (right). RNA-seq (B) and qRT-PCR (C) revealed transcriptional profiles of putative zinc transporter genes in WT and Mzur under low- and high-zinc conditions. Results from three biological replicates are shown. Subcellular locations of these putative zinc transporter proteins are shown on the right of panel B. (D) qRT-PCR analysis of *znuA* transcription in wild-type CCBAU45436 and Mzur grown under conditions of low or high zinc. qRT-PCR analysis of transcription of zinc transporter genes under high levels of zinc (E) and cobalt (200 μM CoCl_2_) (H). qRT-PCR analysis of *c06450* (F) and *znuA* (G) genes in CCBAU45436 in the presence of 200 μM metals (FeCl_3_, CoCl_2_, NiSO_4_, or ZnSO_4_) or 25 μM EDTA. Mean transcription values were compared with that in the medium with EDTA. (C to H) Values are given as means ± SDs from biological triplicates in three independent experiments. *, *P* < 0.05; ***, *P* < 0.001 by *t* test.

RNA-seq analysis revealed that 1,115 and 957 genes were up- and downregulated, respectively, in CCBAU45436 under low-zinc conditions (4 μM TPEN) compared to expression in cells with a high zinc supply (200 μM ZnSO_4_) (Fig. 2A and Table S1A). To determine the regulon of Zur, these transcriptional profiles were compared with those of an in-frame deletion mutant of *zur* under the same conditions (Mzur) (Fig. 2A and Table S1A). Mzur showed 86 and 20 differentially expressed genes under high- and low-zinc conditions, respectively, compared with expression in CCBAU45436. Notably, four genes encoding putative components of metal transporters were upregulated both in CCBAU45436 under low-zinc conditions (log2R ranged from 5.6 to 8.6) and in Mzur under high-zinc conditions (log2R ranged from 3.8 to 8.1) compared to that in CCBAU45436 under high-zinc conditions (Fig. 2B and Table S1A). They are *znuA*, *znuB*, *znuC*, and *c06450*, the last of which encodes a putative periplasmic protein (COG5266) of an ABC-type Co^2+^ transport system (Fig. 2B). Further qRT-PCR experiments confirmed the upregulation of *znuA* and *c06450* under low-zinc conditions (Fig. 2C) and the Zur-dependent repression of *znuA* (Fig. 2D), *znuC-znuB-zur* operon, and *c06450* (Fig. 2E) under high-zinc conditions.

10.1128/mBio.03193-19.9 TABLE S1 RNA-seq analysis of Zur regulon and predicted Zur-box. (A) RNA-seq analysis of Sinorhizobium fredii CCBAU45436 and its Mzur mutant under zinc-replete (high_zinc) and zinc-deplete (TPEN) conditions. (B) Zur-box identified in the genome of Sinorhizobium fredii CCBAU45436 and transcription profiles of associated genes in RNA-seq analysis. Download Table S1, XLSX file, 2.9 MB. Copyright © 2020 Zhang et al. 2020 Zhang et al. This content is distributed under the terms of the Creative Commons Attribution 4.0 International license.

In the genome of CCBAU45436, there are two genes encoding low-affinity zinc transporters of the ZIP family, but no homologs of *troCBA* from A. tumefaciens were found. Conserved regions of ZIP homologs are present in Zip1 (b54490) and Zip2 (d66670) (see Fig. S2). The *zip1* gene was actively transcribed under high-zinc conditions (reads per kilobase per million mapped reads [RPKM] = 916), and showed an even higher transcription level under zinc depletion conditions (log2R = 2.7) as revealed by RNA-seq, but this induction was not dependent on Zur (log2R = 0.15) (Table S1A) and could not be detected by qRT-PCR (Fig. 2C and E). The *zip2* gene was constitutively transcribed at a rather low level under the tested conditions (RPKM < 30) (Fig. 2B and C) and was not regulated by Zur (Fig. 2B and E).

10.1128/mBio.03193-19.2 FIG S2 Putative zinc uptake transporters encodedby the multipartite genome of *S. fredii* CCBAU45436. (A) Genes encoding putative transporters ZnuABC, Zip1, and Zip2 are shown in black. These genes are located on three of the five replicons of CCBAU45436: cSF45436 (chromosome), pSF45436b (chromid), and pSF45436d (an accessory plasmid). (B) Zip1 and Zip2 of CCBAU45436 harbor two characterized conserved regions (shaded) in two transmembrane domains of ZIP family proteins. The partial protein sequences of Zip1 (SF45436_b54490) and Zip2 (SF45436_d66670) were aligned with ZIP protein homologs from Arabidopsis thaliana, Saccharomyces cerevisiae, Glycine max, and Escherichia coli. Download FIG S2, PDF file, 0.2 MB. Copyright © 2020 Zhang et al. 2020 Zhang et al. This content is distributed under the terms of the Creative Commons Attribution 4.0 International license.

It is intriguing that *c06450* encodes a putative periplasmic component of a Co^2+^ transport system responding to zinc depletion, while *znuA* encodes a periplasmic component of a Zn^2+^ transport system (COG4531) (Fig. 2B). To test the specificity of c06450 and ZnuA, qRT-PCR was used to determine transcriptional responses of *znuA* and *c06450* to Co^2+^, Zn^2+^, Fe^3+^, and Ni^2+^ (200 μM), with 25 μM EDTA as a control. High concentrations of both Co^2+^ and Zn^2+^ exerted significant repression on transcription of *c06450* and *znuA* (Fig. 2F and G) (*P* < 0.05) compared to that with EDTA treatment, though the strongest repression was mediated by Zn^2+^. The repression of these two genes by replete Co^2+^ was significantly relieved in Mzur compared to that in CCBAU45436 (Fig. 2H), while *zip1* and *zip2* were not regulated by Zur under the same conditions. Therefore, *c06450* and *znuA* can respond to both zinc and cobalt in a Zur-dependent manner.

**FIG 3:**
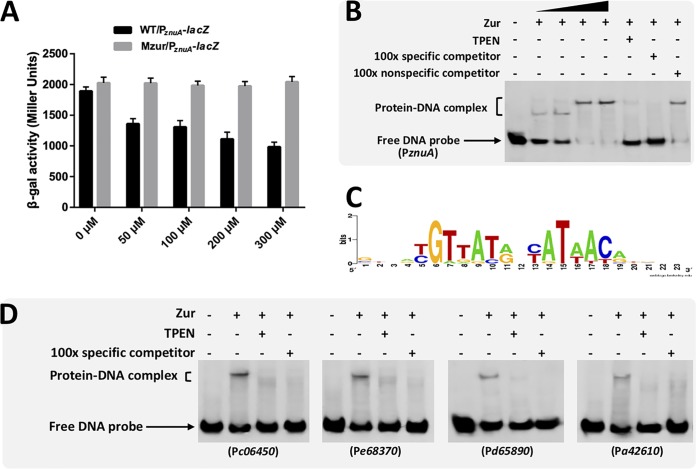
Dose-dependent regulation of *znuA* by zinc and Zur, and EMSA validation of Zur-binding sites. (A) β-galactosidase (β-Gal) assays showing expression of *znuA* promoter*-lacZ* translational fusion in wild-type CCBAU45436 and Mzur grown in different concentrations of ZnSO_4_. (B) Zur binds the *znuA* promoter in a zinc-dependent manner. Biotin-labeled *znuA* promoter fragment was incubated with increasing concentrations (0.33, 0.66, 1.32, and 2.64 μM) of purified Zur of CCBAU45436. (C) DNA sequence logos derived from 17 predicted Zur-binding sites with a score of >5 (Table S1B). The consensus sequence contains a 15-bp palindromic motif (7-1-7 inverted repeat). (D) Biotin-labeled promoter fragment containing predicted Zur-binding sites was incubated with purified Zur (2.64 μM). For panels B and D, 200 μM ZnSO_4_ was present in the reaction buffer of all samples. + and − indicate the presence and absence, respectively, of Zur protein, a 100-fold excess of unlabeled *znuA* promoter fragment (specific competitor), unlabeled mutated *znuA* promoter DNA fragment (nonspecific competitor), or 50 μM TPEN (zinc chelator).

Further analysis using a translational P*_znuA_-lacZ* fusion revealed that expression of ZnuA was unchanged in Mzur under zinc-replete conditions (50 to 300 μM ZnSO_4_) (Fig. 3A), while the same fusion in the wild-type CCBAU45436 showed a stepwise decreased expression pattern with increasing ZnSO_4_ levels in the medium. This is in line with the decreased Zur expression upon elevated zinc level (Fig. 1B). To test whether Zur can directly bind the putative Zur box of the *znuA* promoter, an electrophoretic mobility shift assay (EMSA) with purified His_6_-Zur protein was performed. When the concentration of purified His_6_-Zur protein was increased (above 1.32 μM), the *znuA* promoter probe showed a typical gel shift pattern of oligomeric Zur binding compared with the pattern observed with a lower concentration of Zur (Fig. 3B). When the Zn^2+^ chelator TPEN was present, the amount of retarded DNA fragment was significantly reduced, indicating a zinc-dependent binding of Zur to the *znuA* promoter.

Bioinformatics analyses further identified a consensus sequence for the Zur box containing a 15-bp palindromic motif (7-1-7 inverted repeat) and found 17 binding sites in the genome of CCBAU45436 (Fig. 3C; Table S1B). In addition to binding sites within promoter regions of *znuC* and *znuA*, a binding box was found in the promoter region of *c06450*. No binding sites were found around the coding regions of *zip1* and *zip2*. The other fourteen putative Zur targets included transcriptional regulators belonging to the LuxR and MerR families and components of transport systems for capsular polysaccharide, lipopolysaccharide, and l-proline/glycine/betaine (Table S1B). Four representative Zur boxes (those associated with *c06450*, *e68370*, *d65890*, and *a42610*) were further verified by EMSA. All four tested fragments were retarded in the EMSA when the Zn^2+^ chelator TPEN was absent (Fig. 3D). However, not all genes with a Zur box were differentially expressed under test conditions (Table S1B), indicating the involvement of other transcriptional factors in regulating these target genes. Zur-dependent regulation was only detected under high-zinc conditions for five boxes associated with *znuA*, *znuC-znuB-zur*, *c06450*, *c06460* (encoding a glutathione *S*-transferase), and *d65890* (encoding a chemotaxis protein methyltransferase CheR) (Table S1B).

### ZnuABC is the predominant zinc uptake transporter under zinc-deplete conditions.

Although *c06450* was annotated as a putative cobalt transport protein, metal uptake experiments showed that *znu* rather than *c06450* played a more important role in uptake of both cobalt and zinc (see Fig. S3). The contribution of Zip1 and Zip2 was also limited under test conditions. To further determine the relative importance of *znuA*, *c06450*, *zip1*, and *zip2* under zinc-deplete conditions, individual and combined mutants of these genes were inoculated in equal quantities onto plates with the M9 minimal medium with or without 50 μM ZnSO_4_ (see Fig. S4). Colonies were rarely observed on the M9 minimal medium (10^−1^ to 10^−5^ dilutions) without supplementing ZnSO_4_ for the *znuA* mutant (MznuA) and its derived mutants lacking both *znuA* and other putative zinc transporter genes (MznuAzip1, MznuAzip2, MznuAc06450, MznuAczip1zip2, and MznuAc06450zip1zip2). No defects were found for the other mutants harboring intact *znuABC* genes (Mzur, Mzip1, Mzip2, Mzip1zip2, and Mc06450). The growth defects of mutants lacking the *znuA* gene were restored either by adding 50 μM ZnSO_4_ or by introducing the complementary vector pB-*znuA* (but not pB-*zip1* or pB-*c06450*) into corresponding mutants. On the other hand, 2 μM CoCl_2_ partially restored the growth of mutants lacking *znuA* on M9 plates, and 20 μM CoCl_2_ further improved their growth to a level similar to that of CCBAU45436 (Fig. S4). No difference was observed between MznuA and the other combined mutants lacking *znuA*, and 20 μM CoCl_2_ itself reduced the growth of CCBAU45436 on M9 plates. When the growth curves of representative strains were monitored in liquid M9 medium (Fig. S3), a more severe growth defect than that of MznuA was observed for the combined mutants lacking *znuA* and at least one more gene among *zip1*, *zip2*, and *c06450*. A replete supply of ZnSO_4_ restored their growth rate (Fig. S3). When all mutants lacking *znuA* were grown on the rich medium, yeast-mannitol-agar (YMA), no significant growth defects were found (Fig. S4). However, the mutants without the *znuA* gene were sensitive to supplemented 50 μM EDTA, which can sequester metal ions in the YMA medium. Introducing a functional *znuA* carried by pB-*znuA* into corresponding *znuA*-lacking mutants can restore this growth defect. The sequestration effect of EDTA can be released by adding 50 μM ZnSO_4_ (Fig. S4). Therefore, ZnuA is critical for adaptation to zinc-deplete conditions, while the contribution by Zip1, Zip2, and c06450 can only be detected under certain conditions.

10.1128/mBio.03193-19.3 FIG S3 Znu as the major zinc uptake system under zinc-deplete conditions. (A) Intracellular cobalt content of strains grown in M9 medium supplied with 10 μM CoCl_2_. (B andC) Intracellular zinc content of strains grown in M9 medium supplied with 10 μM ZnSO_4_. (D to G) Growth curves determined in M9 medium without (C and F) or with (D and G) 100 μM ZnSO_4_. Values are mean ± SDs from biological triplicates. Different lowerletters indicate significant difference (Duncan test, alpha = 0.05). Download FIG S3, PDF file, 0.5 MB. Copyright © 2020 Zhang et al. 2020 Zhang et al. This content is distributed under the terms of the Creative Commons Attribution 4.0 International license.

10.1128/mBio.03193-19.4 FIG S4 Znu is essential for low zinc adaptation. (A) Cell suspension at an OD_600_ of 1.0 was serially diluted and spotted onto plates with the M9 minimal medium with or without 50 μM ZnSO_4_ or onto plates with the YMA medium supplemented with 50 μM EDTA or 50 μM EDTA and 50 μM ZnSO_4_. Plasmids pB-*znuA*, pB-*zip1*, and pB-*c06450* were used in complementation experiments. (B) Growth defects of *znu* mutants on M9 plates was partially restored by adding CoCl_2_. Cell suspension at an OD_600_ of 1.0 was serially diluted and spotted onto plates with the M9 medium supplemented with 20 μM or 2 μM CoCl_2_. Download FIG S4, PDF file, 1.1 MB. Copyright © 2020 Zhang et al. 2020 Zhang et al. This content is distributed under the terms of the Creative Commons Attribution 4.0 International license.

MznuB, MznuC, and MznuAznuB exhibited delayed growth curves similar to that of MznuA in liquid M9 medium compared to that of CCBAU45436 (Fig. S3). Mc06450znuB, MznuAc06450znuB, and MznuAc06450zip1zip2znuB showed similar growth curves, with a reduced optical density at 600 nm (OD_600_) at the stationary phase compared to that of mutants lacking just *znu* genes (MznuA, MznuB, MznuC, and MznuAznuB) (Fig. S3). These results imply that ZnuA is the major periplasmic zinc binding protein to deliver zinc to ZnuBC under the test conditions.

### Zinc uptake systems make cumulative contributions to symbiotic adaptations.

**FIG 4:**
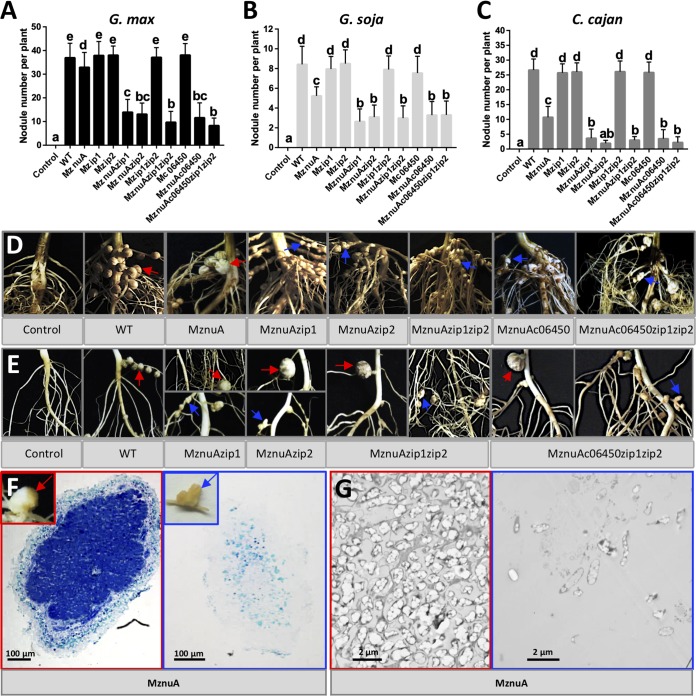
Impaired infection ability of mutants lacking *znu.* Number of infected nodules per plant of *G. max* (A), *G. soja* (B) and *C. cajan* (C). Means ± SDs are based on more than 20 to 40 scored plants from multiple independent experiments. Different lowercase letters indicate significant difference (Duncan test, alpha = 0.05). Representative photos showing infected nodules (red arrows) and bumps (blue arrows) observed on roots of *G. max* (D), *G. soja* (E), and *C. cajan* (F) inoculated with corresponding strains. Light microscopy pictures (F) and transmission election microscopy pictures (G) of sections for infected nodules (red border) and bumps (blue border) on *C. cajan* inoculated with MznuA. Bumps were rarely infected by rhizobia.

MznuA and other tested mutants lacking *znu* genes (MznuAzip1, MznuAzip2, MznuAzip1zip2, MznuAc06450, MznuAc06450zip1zip2, MznuB, MznuC, MznuAznuB, Mc06450znuB, MznuAc06450znuB, and MznuAc06450zip1zip2znuB) induced fewer nodules on Glycine max C08, *Glycine soja* W05, and Cajanus cajan than CCBAU45436 and mutants harboring intact *zunABC* genes (Mzip1, Mzip2, Mzip1zip2, and Mc06450) (Fig. 4A to C; see also Fig. S5) (Duncan’s test, alpha = 0.05). Notably, plants of three legume species inoculated with MznuAzip1, MznuAzip2, MznuAzip1zip2, MznuAc06450, MznuAc06450zip1zip2, MznuB, MznuC, MznuAznuB, Mc06450znuB, MznuAc06450znuB, and MznuAc06450zip1zip2znuB had fewer nodules than plants inoculated with MznuA (Fig. 4A to C; Fig. S5) (Duncan’s test, alpha = 0.05), indicating cumulative contributions of *znuA*, *zip1*, *zip2*, and *c06450* genes and that ZnuBC may also receive zinc from sources other than ZnuA during symbiosis.

10.1128/mBio.03193-19.5 FIG S5 Comparative analysis of nodulation phenotypes of mutants lacking different *znu* genes. (A) Representative photos showing infected nodules (red arrows) and bumps (blue arrows) observed on roots of *G. max* and *G. soja* inoculated with corresponding strains. Numbers of infected nodules per plant of *G. max* (B), *G. soja* (C), and *C. cajan* (D). Means ± SDs are based on 20 to 30 scored plants from three independent experiments. Different lowercase letters indicate significant difference (Duncan test, alpha = 0.05). Numbers of infected nodules per plant of *G. max* (E), *G. soja* (F), and *C. cajan* (G) inoculated with corresponding strains under conditions with or without 700 μM ZnSO_4_ in vermiculite moistened with low-N nutrient solution. Means ± SDs are based on 20 to 30 scored plants from three independent experiments. **, *P* < 0.01; ***, *P* < 0.001 by *t* test. Download FIG S5, PDF file, 2.2 MB. Copyright © 2020 Zhang et al. 2020 Zhang et al. This content is distributed under the terms of the Creative Commons Attribution 4.0 International license.

Those mutants that showed more severe symbiotic defects than MznuA induced many bumps on *G. max* and *G. soja* roots (Fig. 4D and E; Fig. S5). Root bumps were frequently observed on *C. cajan* roots inoculated with MznuA (Fig. 4F) and other combined mutants lacking *znu* genes. As shown in light microscopy (Fig. 4F) and transmission electron microscopy (Fig. 4G) photos, these bumps were poorly infected compared with nodules induced by the same mutant. In contrast to *G. max* plants, these bumps were not found in all *G. soja* and *C. cajan* plants inoculated with corresponding mutants (Fig. 4D to F), possibly due to intrinsic genetic variation in seeds of these two species. On the other hand, for those nodules of *G. max*, *G. soja*, and *C. cajan* induced by the mutants lacking the *znuA* gene, most of them were well infected except a small number of *G. max* nodules that harbored a clearly reduced number of intracellular bacteroids (see Fig. S6). Shoot dry weights of *G. max* and *C. cajan*, but not *G. soja*, inoculated with the mutants lacking *znu* genes were significantly lower than those of plants inoculated with the other strains tested (see Fig. S7) (Duncan’s test, alpha = 0.05).

10.1128/mBio.03193-19.6 FIG S6 Ultrathin sections of *G. max* (A), *G. soja* (B), and *C. cajan* (C) nodules. Pictures of ultrathin sections of 40-day postinoculation (dpi) nodules infected by CCBAU45436 and representative mutants were obtained by transmission electron microscopy. Download FIG S6, PDF file, 1.1 MB. Copyright © 2020 Zhang et al. 2020 Zhang et al. This content is distributed under the terms of the Creative Commons Attribution 4.0 International license.

10.1128/mBio.03193-19.7 FIG S7 Symbiotic performance of mutants lacking individual or multiple zinc transporter genes. Shoot dry weight of *G. max* (A), *G. soja* (B), and *C. cajan* (C) plants inoculated with *S. fredii* CCBAU45436 and its derivatives lacking individual or multiple zinc transporter genes. (D) Shoot dry weight of *G. soja* plants inoculated with CCBAU25509/CCBAU05684/CCBAU05631 and their *znuA* mutants. Means ± SDs are based on 20 to 30 scored plants from three independent experiments. Different lowercase letters indicate significant difference (Duncan test, alpha = 0.05). ***, *P* < 0.001 by *t* test of the mean comparison with that of wild-type *S. fredii* CCBAU25509, *S. sojae* CCBAU05684, or *Sinorhizobium* sp. CCBAU05631; ns, nonsignificant. Download FIG S7, PDF file, 0.3 MB. Copyright © 2020 Zhang et al. 2020 Zhang et al. This content is distributed under the terms of the Creative Commons Attribution 4.0 International license.

**FIG 5:**
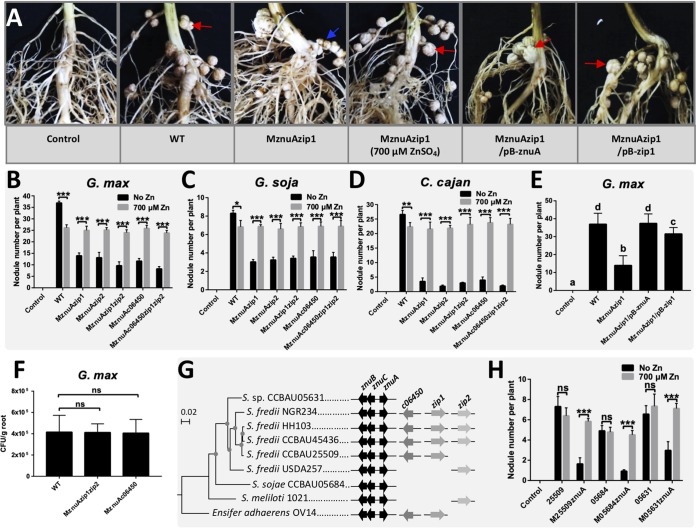
Replete zinc recovers nodulation defects of mutants lacking *znu*. (A) Representative photos showing infected nodules (red arrows) and bumps (blue arrows) observed on roots of *G. max* inoculated with corresponding strains with or without supplement of 700 μM ZnSO_4_ in vermiculite. pB-*znuA* and pB-*zip1* carrying functional *znuA* and *zip1*, respectively, were used in complementary experiments. Numbers of infected nodules per plant of *G. max* (B), *G. soja* (C), and *C. cajan* (D) inoculated with corresponding strains under conditions with or without 700 μM ZnSO_4_ in vermiculite moistened with low-N nutrient solution. (E) Number of infected nodules per plant of *G. max* inoculated with MznuAzip1 and its derivatives carrying pB-*znuA* or pB-*zip1* on *G. max*. (F) Root surface colonization by CCBAU45436, MznuAzip1zip2, and MznuAc06450 on *G. max*. CFU were counted 5 days after plating the suspension of cells collected by using ultrasound. (G) Maximum likelihood phylogenic tree based on 2,166 core genes of representative *Sinorhizobium* strains. The filled circles on the branches indicate >95% bootstrap support. The scale bar represents 0.02 nucleotide substitution per site. The presence of conserved zinc transporter genes in the corresponding genome is indicated. (H) Numbers of infected nodules per plant of *G. soja* inoculated with CCBAU25509, CCBAU05684, CCBAU05631, and their *znuA-*insertion mutants under conditions supplemented with or without 700 μM ZnSO_4_ in vermiculite moistened with low-N nutrient solution. (B to F and H) Means ± SDs are based on more than 20 to 40 scored plants from multiple independent experiments. ns, *P* > 0.05; *, *P* < 0.05; **, *P* < 0.01; ***, *P* < 0.001 by *t* test.

The reduced number of nodules induced on *G. max*, *G. soja*, and *C. cajan* by mutants lacking intact *znuABC* (MznuAzip1, MznuAzip2, MznuAzip1zip2, MznuAc06450, MznuAc06450zip1zip2, MznuA, MznuB, MznuC, MznuAznuB, Mc06450znuB, MznuAc06450znuB, and MznuAc06450zip1zip2znuB) were largely rescued by adding 700 μM ZnSO_4_ to the growth medium, which was vermiculite moistened with low-N nutrient solution (Fig. 5A to D; Fig. S5), but not by adding cobalt (see Fig. S8). Moreover, the root bumps induced by various mutants, such as those on *G. max* plants inoculated with MznuAzip1, were not observed when 700 μM ZnSO_4_ was added to the vermiculite (Fig. 5A). Symbiotic defects of MznuAzip1 were fully and partially restored by introducing pB-*znuA* and pB-*zip1*, respectively (Fig. 5E). Further analysis revealed that the numbers of CFU of mutants such as MznuAzip1zip2 and MznuAc06450 on the rhizoplane of *G. max* were indistinguishable from those of the wild-type CCBAU45436 (Fig. 5F). This suggests that these mutants have similar survival rates on the root surface as the wild-type strain. The symbiotic performance of Mzur was indistinguishable from that of the wild-type CCBAU45436 regarding nodule number and shoot dry weight on *G. max*, *G. soja*, and *C. cajan*.

10.1128/mBio.03193-19.8 FIG S8 Nodulation defects of mutants lacking *znu* cannot be recovered by replete cobalt. Nodule number per plant of Glycine max and *Glycine soja* were recorded under conditions with different concentrations of cobalt being supplied. Means ± SDs are based on more than 20 to 30 scored plants. ns, *P* > 0.05; ***, *P* < 0.001 by *t* test. There were no nodules formed on Cajanus cajan plants inoculated with either WT or the mutant when cobalt was added. Download FIG S8, PDF file, 0.1 MB. Copyright © 2020 Zhang et al. 2020 Zhang et al. This content is distributed under the terms of the Creative Commons Attribution 4.0 International license.

### The contribution of *znu* to symbiotic adaptation is lineage dependent.

Comparative genomics analysis showed that *znuABC* orthologous genes are conserved in *Sinorhizobium*, while *zip1*, *zip2*, and *c06450* are accessory genes with limited phyletic distribution (Fig. 5G). For example, *S. fredii* CCBAU25509 lacks *zip2*, and Sinorhizobium sojae CCBAU05684 and *Sinorhizobium* sp. CCBAU05631 do not contain *zip1*, *zip2*, or *c06450*, though these three strains and CCBAU45436 are all effective microsymbionts of *G. soja* W05. The *znuA* mutants of CCBAU25509, CCBAU05684, and CCBAU05631 formed a reduced number of nodules on *G. soja* W05 than the wild-type strains and the *znuA* mutant of CCBAU45436 (Fig. 5H and 4B), indicating an important role of the *znuA* gene in modulating nodulation of all tested strains on *G. soja* W05. These nodulation defects can be restored by adding replete ZnSO_4_ to the vermiculite (Fig. 5H).

Shoot dry weights of *G. soja* W05 plants inoculated with the *znuA* mutant of CCBAU25509 or CCBAU05684 were significantly lower than those of plants inoculated with the wild-type strains (Fig. S7) (*P* < 0.001), and these two *znuA* mutants induced root bumps on *G. soja* plants. These phenomena were not observed for the *znuA* mutants of CCBAU45436 and CCBAU05631 (Fig. S7). Therefore, despite the fact that CCBAU05631 lacks *zip1*, *zip2*, and *c06450* as CCBAU05684 does, the symbiotic defect of the *znuA* mutant of CCBAU05631 was not as severe as that of the *znuA* mutant of CCBAU05684, implying differential integrations of Znu with different genomic backgrounds.

### Zinc uptake systems are required for proper induction of nodulation and T3SS genes.

**FIG 6:**
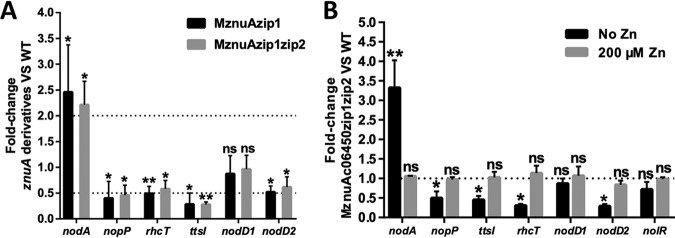
Transcription of rhizobial *nod* and T3SS genes. (A) qRT-PCR analysis of *nod* and T3SS gene transcription in wild-type CCBAU45436 and MznuAzip1 or MznuAzip1zip2 mutants grown in M9 medium with 12-h induction by 1 μM genistein. (B) qRT-PCR analysis of *nod* and T3SS genes in wild-type CCBAU45436 and MznuAc06450zip1zip2 grown in M9 medium (with or without 200 μM ZnSO_4_) with 12-h induction by 1 μM genistein. Mean transcription values were compared with the wild-type CCBAU45436. ns, *P* > 0.05; *, *P* < 0.05; **, *P* < 0.01 by *t* test. Values are means ± SDs from biological triplicates in three independent experiments.

The root bumps of *G. max* plants inoculated with mutants such as MznuAzip1, MznuAzip1zip2, and MznuAc06450zip1zip2 indicated potential disturbance of symbiotic signaling. qRT-PCR analysis of free-living cultures in the presence of symbiotic signal genistein (Fig. 6A and B) revealed that *nodD2* (encoding a transcriptional repressor of *nod* genes) rather than *nodD1* (encoding a transcriptional activator of *nod* genes) was downregulated in MznuAzip1, MznuAzip1zip2, and MznuAc06450zip1zip2 compared with that in CCBAU45436 (*P* < 0.05). The *nodA* gene, one of the core *nod* genes, was upregulated in these mutants (*P* < 0.05), while *ttsI* encoding the transcriptional activator of type 3 secretion system (T3SS) genes, the T3SS component gene *rhcT*, and the effector gene *nopP* were downregulated (*P* < 0.05). These differential transcriptional profiles were restored by supplying replete zinc, as shown for MznuAc06450zip1zip2 (Fig. 6B), indicating that proper induction of nodulation and T3SS genes requires ample zinc.

## DISCUSSION

Efficient zinc uptake is critical for diverse pathogens to infect eukaryotes, but the role of zinc homeostasis in symbiosis is poorly understood (18, 30). A notable symbiotic defect of *znu*-derived mutants of CCBAU45436 was the reduced number of nodules on *G. max*, *G. soja*, and *C. cajan* plants. This phenomenon was also observed for the *znuA* mutants of *S. fredii* CCBAU25509, *S. sojae* CCBAU05684, and *Sinorhizobium* sp. CCBAU05631 on *G. soja*. Moreover, poorly infected pseudonodules can be frequently found on host roots inoculated with these *znu*-derived mutants. This could be due to the downregulation of *nodD2*, *ttsI*, T3SS genes, and *nopP* in *znu*-derived mutants. This view is supported by several lines of evidence as described below. The poorly infected root bumps are commonly observed on soybean roots inoculated with incompatible *Sinorhizobium* and *Bradyrhizobium* strains (25, 31–33). It has been demonstrated that soybean allelic genes *Rj2* and *Rfg1* encoding the Toll-interleukin receptor/nucleotide-binding site/leucine-rich repeat class of plant resistance proteins are involved in restricting nodulation by certain rhizobia (34). In the presence of *rj2* (*Rfg1*) soybean cultivar JD17, compatible *Sinorhizobium* clones, with their T3SS gene cluster including *nopP* disrupted by indigenous mobile insertion sequences, can be selected by the host from the inocula of incompatible ancestral strains, including CCBAU05631, CCBAU25509, and *S. fredii* CCBAU83666 (25). By swapping *nopP* between CCBAU45436 and CCBAU25509, it was further demonstrated that *nopP*_45436_ from CCBAU45436 enabled CCBAU25509 to nodulate the *rj2* (*Rfg1*) soybean C08, while CCBAU45436 carrying *nopP*_25509_ did not nodulate C08 (35). These studies highlight that natural variation in NopP from different *Sinorhizobium* strains can modulate symbiotic compatibility with soybeans and that NopP from CCBAU45436 is a compatible effector with *rj2* (*Rfg1*) soybeans. Similarly, natural variation of NopP sequences was found in *Bradyrhizobium* and determines bradyrhizobial incompatibility with *Rj2* soybeans, in which the plant defense marker gene *PR-2* was activated by incompatible strains (33). Therefore, NopP can be one of the key effectors modulating the compatibility of rhizobia associated with different genotypes of soybeans. Moreover, the secretion of NopP by *S. fredii* NGR234 and its phosphorylation by plant kinase from diverse host legumes have been demonstrated (36, 37). The mutation of *nopP* in NGR234 can have a negative, positive, or little effect on the nodule number on different legume species (36, 37), possibly due to the diverse immunity systems of different legumes. These findings suggest that the formation of poorly infected root bumps and reduced number of infected nodules by *znu*-derived mutants may be mediated by effector-triggered immunity.

TtsI is the transcriptional activator of T3SS genes and effector genes, including *nopP*, and can be transcriptionally induced by the NodD1-flavonoid complex (38, 39), which can also activate the transcription of *nod* genes involved in the biosynthesis of Nod factors (NFs). Although rhizobial NFs are the key symbiotic signal initiating both infection and nodule organogenesis (40), it was recently demonstrated that excess amounts of NFs negatively regulate the initiation of infection threads in *M. truncatula*, and NF hydrolase MtNFH1 is involved (41). The abnormal branching of indeterminate nodules, as seen on MtNFH1-deficient *M. truncatula* plants, was also observed on *C. cajan* roots inoculated with mutants lacking *znu* genes of CCBAU45436 in this study. It should be noted that *nodA*, one of the core *nod* genes, was upregulated in MznuAzip1, MznuAzip1zip2, and MznuAc06450zip1zip2 in the presence of soybean flavonoid genistein, while *nodD2* rather than *nodD1* was downregulated. There is evidence that *nodD2* in Bradyrhizobium japonicum and *S. fredii* strains nodulating soybeans can repress the transcription of *nod* genes (42, 43). In *S. fredii* NGR234, NodD2 can repress the transcription of *nodABC* genes, and the *nodD2* mutant formed bacteria-free pseudonodules on Lablab purpureus, Pachyrhizus tuberosus, Psophocarpus palustris, Tephrosia vogelii, Crotalaria juncea, and Flemingia congesta, and Fix^−^ nodules on Vigna unguiculata and *C. cajan* (38, 44). In *S. fredii* HH103, a *nolR* mutant with a higher level of NF production formed significantly fewer nodules on *G. max* than the wild-type strain (45), though this gene was not differentially transcribed in MznuAc06450zip1zip2. Therefore, the downregulation of *nodD2* and upregulation of *nod* genes involved in NF biosynthesis in these mutants in the presence of genistein are in line with the impaired infection and nodulation ability.

Cumulative contributions of c06450, Zip1, and Zip2 to Znu-dependent regulation of infection and nodulation were observed, and symbiotic defects of mutants lacking *znu* genes can be recovered by a replete supply of zinc. ZIP, found in all kingdoms of life, can mediate metal uptake into the cytosol (46). Zip1 and Zip2 are accessory proteins in *Sinorhizobium* and were not regulated by Zur in CCBAU45436. Both of them were required for the Znu-dependent best symbiotic performance of CCBAU45436. Similarly, a constitutively expressed ZIP protein ZupT and the conserved ZnuABC in Salmonella enterica are required for maximum virulence in mice and the infection rate of the *znu-zup* double mutant was significantly lower than the *znu* mutant (9). Although bacterial and plant ZIPs have been reported to be relatively nonselective, promoting the uptake of Zn^2+^, Fe^2+^, Co^2+^, and Mn^2+^ in E. coli and Zn^2+^, Fe^2+^ and Cd^2+^ in plants (46), adding zinc to the rhizosphere of legumes can restore the effective symbiotic performance of MznuAzip1, MznuAzip2, and MznuAzip1zip2. Similarly, the more severe symbiotic defect of MznuAc06450 and MznuAc06450zip1zip2 than MznuA can be overcome by adding zinc to the legume rhizosphere. c06450 is an accessory periplasmic metal-binding protein and was highly induced under zinc-deplete conditions. It has been reported that a periplasmic zinc-binding protein ZinT may deliver Zn^2+^ to ZnuA in E. coli and S. enterica and contribute to Znu-dependent bacterial infection (16, 17, 21). Therefore, zinc restriction by host (18) is a common nutritional immunity faced by both pathogenic and symbiotic bacteria.

In addition to *znuA* and *znuC-znuB-zur*, conserved in many other bacteria (6, 10–16), accessory *c06450* was directly repressed by the canonical zinc-sensing transcriptional factor Zur. Similar to Zn^2+^, replete Co^2+^ led to Zur-dependent repression of *znu* operons and *c06450*. This is consistent with the ability of Co^2+^ to bind the Zur dimer in its four exchangeable Zn^2+^ sites *in vitro* and to promote Zur binding to the promoter of *znuA* in *Salmonella* (47). This phenomenon can be explained as cobalt mismetalation observed under inhibitory concentration of cobalt for *Salmonella* (47) and for *Sinorhizobium* strains in this study. Moreover, a higher binding affinity of Zur to Zn^2+^ than to Co^2+^ has been demonstrated *in vitro* (47), and adding Co^2+^ to the *G. max* rhizosphere failed to restore the nodulation defect of MznuAc06450zip1zip2.

In contrast to mutants lacking *znu* genes, symbiotic performance of Mzur was not impaired despite its reduced competitive nodulation ability (48). This can largely be explained by the observation that the high-affinity zinc transporter genes *znuABC* and *c06450* were upregulated in Mzur. Moreover, transcriptomics analysis of the *zur* mutant and the wild-type CCBAU45436 demonstrated that Zur is a local regulator for a small subset of the zinc regulon (4.2%). This is consistent with the small Zur regulon of other bacteria such as Neisseria meningitidis (49). Notably, some zinc-responding genes are within the list of direct targets of Zur but did not show a Zur-dependent transcriptional profile under test conditions, indicating the coordinated transcriptional regulation by other factors. Alternatively, this could be due to the fact that dimeric or oligomeric Zur formed under different zinc levels can exert differential regulation on target genes through extension of its DNA-binding footprint (50). Oligomeric Zur binding of DNA *in vitro* can also be observed with increased concentration of purified Zur from Streptomyces coelicolor (50) and, in this study, *S. fredii*. Although bacteria have a low fraction of zinc proteins (4.9%) on average, these proteins have diverse functions such as enzyme activity and roles in transcription, storage transport, and signal transduction (51). Consequently, as many as 2,072 differentially expressed genes between zinc-deplete and -replete conditions were identified herein for CCBAU45436, indicating a cascade regulation of almost one-third of the genome by zinc under the tested free-living conditions. It can be deduced that zinc depletion in mutants lacking *znu* genes may lead to a global effect on cellular physiology. When the symbiotic signal genistein from soybean was present (52), the transcription of key symbiosis genes such as *nodD2*, *nodA*, *ttsI*, *nopP*, and T3SS genes was altered in mutants lacking multiple zinc transport components compared to that in the wild-type CCBAU45436. In the presence of replete zinc, these transcriptional changes were restored to the level of the wild-type strain. Therefore, zinc homeostasis modulated by zinc transporters is crucial for optimizing the transcription profiles of key genes involved in early symbiotic interaction with legume hosts, though the mechanisms underlying this link remain elusive. Notably, in the mutant of *mucR1* lacking a zinc-finger transcriptional regulator, *nodD2* was significantly upregulated compared with that in the wild-type CCBAU45436 under symbiotic conditions with soybean (27). MucR1 has a conserved C_2_H_2_ zinc coordination sphere essential for its DNA-binding activity (53). It should be further investigated how MucR1 and other putative zinc-responding factors are involved in regulating *nod* and/or T3SS genes.

### Conclusions.

**FIG 7:**
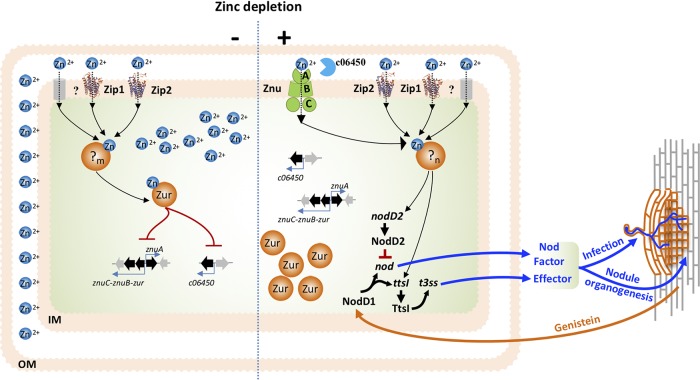
Working model for the role of zinc starvation machinery in modulating symbiotic compatibility. Under zinc replete conditions (−, left), *znu* and *c06450* were repressed by Zur. Zip1, Zip2, and an unknown zinc transporter(s) import zinc. Under zinc-deplete conditions during symbiosis establishment (+, right), transcriptional repression of *znu* and *c06450* by Zur was released due to decreased intracellular zinc level. Znu together with accessory c06450/Zip1/Zip2 and an unknown zinc transporter(s) in the *Sinorhizobium* pangenome were essential for proper transcription of T3SS genes and *nodD2*, which are involved in regulating symbiotic compatibility during both infection and nodule organogenesis. IM, inner membrane; OM, outer membrane; ?_m_ and ?_n_ indicate that pools of zinc-associated proteins under zinc-replete and -deplete conditions, respectively, may differ as implied by the transcriptomes.

In this study, we show that *znuABC* and *c06450* were directly repressed by the zinc-dependent transcriptional regulator Zur in *S. fredii* CCBAU45436 under zinc-replete conditions and were derepressed under zinc-deplete conditions. These zinc starvation-responsive genes were not essential for rhizoplane colonization. However, accessory proteins, including c06450 and low-affinity zinc transporters Zip1 and Zip2, made cumulative contributions to Znu-associated regulation of *Sinorhizobium* compatibility with legume hosts. In this process, rhizobia needed enough zinc to modulate the transcriptional level of nodulation and T3SS genes, which were induced by a symbiotic signal (genistein) from soybean and are well known for their crucial roles in modulating infection and nodulation (Fig. 7). The regulation mechanisms of nodulation and T3SS genes by zinc homeostasis during the establishment of symbiosis remain elusive, considering that around one-third of the genome can be differentially expressed under zinc starvation conditions. In contrast to the elite strain *S. fredii* CCBAU45436, more severe defects in nodulation were observed for the *znuA* mutant of *Sinorhizobium* strains that harbor an incomplete set of *c06450*, *zip1*, and *zip2* in their genomes and are characterized by their limited symbiotic host range and geographical distribution patterns (24–26, 54–56). These findings imply that accessory components in zinc starvation machinery may contribute to the modulation of nutrient immunity and ecological success of rhizobia.

## MATERIALS AND METHODS

### Bacterial strains, plasmids, and growth conditions.

Bacterial strains and plasmids used in this study are listed in Table S2 in the supplemental material. *Sinorhizobium* strains were grown at 28°C in tryptone-yeast extract (TY) medium (57), YMA medium (57), or modified-M9 minimal medium (58). E. coli strains were cultured in Luria-Bertani (LB) medium at 37°C (58). Antibiotics were added at concentrations described earlier (59).

10.1128/mBio.03193-19.10 TABLE S2 Strains, plasmids, and primers used in this study. Download Table S2, XLSX file, 0.02 MB. Copyright © 2020 Zhang et al. 2020 Zhang et al. This content is distributed under the terms of the Creative Commons Attribution 4.0 International license.

### Construction and complementation of *Sinorhizobium* mutants.

The PCR primers and their specific roles in this study are listed and described in Table S2. To construct the marker-free *znuA* deletion mutant MznuA from the Δ*znuA*::*Gm* mutant constructed earlier (27), the gentamicin resistance cassette was excised by introducing, by conjugation, the plasmid pCM157 expressing Cre recombinase (60), and pCM157-cured clones were identified as tetracycline-sensitive ones. To generate Mzur, Mzip1, Mzip2, MznuAzip1, and MznuAzip2, the *cre-lox* system (60) was used for individual deletion of *zur*, *zip1*, and *zip2* in the wild-type CCBAU45436 and the deletion of *zip1* or *zip2* in MznuA. The same procedure described previously was used (59). Briefly, the suicide plasmid pCM351 containing the upstream and downstream flanking regions of each target gene was constructed and subsequently transformed into the recipient strain by triparental conjugation with helper plasmid pRK2013 (61).

Various pVO155 (62) derivatives containing an internal fragment of individual target genes were used to construct insertion mutations of *zip2* in the backgrounds of MznuAzip1 or Mzip1 derived from CCBAU45436 and to generate insertion mutations of *znuA* in CCBAU25509 or CCBAU05684, while a *znuA* mutation in CCBAU05631 was generated by pJQ200SK (62) containing its internal fragment. These genetic manipulations generated MznuAzip1zip2, Mzip1zip2A, M25509znuA, M05684znuA, and M05631znuA.

To generate Mc06450, MznuAc06450, and MznuAzip1zip2c06450, a seamless cloning approach as described earlier (59, 63), using a pJQ200SK derivative harboring two PCR fragments encompassing the upstream and the downstream regions of *c06450*, was used to delete *c06450* in the backgrounds of wild-type CCBAU45436, MznuA, or MznuAzip1zip2. The seamless cloning approach was also used to delete *znuB* in the backgrounds of wild-type CCBAU45436, MznuA, Mc06450, MznuAc06450, or MznuAc06450zip1zip2 and to delete *znuC* in the wild-type CCBAU45436, resulting in MznuB, MznuAznuB, Mc06450znuB, MznuAc06450znuB, MznuAc06450zip1zip2znuB, and MznuC.

CCBAU45436_3×Myc was constructed by introducing a pVO155 derivative containing a fragment amplified using the primers Zur_tag_F/Zur_tag_F. To construct the plasmids expressing *znuA*, *c06450*, or *zip1*, the fragments containing individual coding sequences of CCBAU45436 were amplified and cloned into pBBR1MCS-3 (64). The resultant pBBR1MCS-3 derivatives were then conjugated into corresponding mutants by triparental mating. All constructs were verified by PCR and Sanger sequencing.

### Bioinformatics procedure.

To predict Zur-binding sites, 42 manually curated Zur-binding motifs from *Rhizobiales*, described on the RegPrecise website (65), were used as a training set, and then a weight matrix was constructed using the CONSENSUS algorithm (66) on the Regulatory Sequence Analysis Tools (RSAT) website (67). Then, we used this matrix to perform a genome-wide screening for putative Zur-binding sites in the CCBAU45436 genome. Six hits with scores above ten were added to the training set, resulting in a set of 48 curated Zur-binding motifs. This updated training set was then used for final prediction of Zur-binding sites in CCBAU45436 (score > 5). DNA sequence logos were generated by WebLogo (68). Protein sequence alignment was generated using ClustalW (69). A neighbor-joining phylogenetic tree was constructed using MEGA 7 (70). Protein structure homology modeling for Zip1 and Zip2 was performed on the SWISS-MODEL server (71), using two different Zn^2+^-substituted structures at 2.4 Å as the structural templates, and results are shown in Fig. 2B and Fig. 7. Core genomes of nine *Sinorhizobium* strains were defined by the bidirectional best-hit algorithm as described earlier (54). Two thousand one hundred sixty-six core genes were aligned with ClustalW (69) and trimmed using Gblocks (72). The trimmed alignment was used for the construction of a maximum likelihood phylogenic tree through MEGA 7 (70).

### RNA-seq, Real-time qPCR, reverse transcriptase PCR, and β-galactosidase activity assays.

Early mid-log-phase rhizobial cultures in modified M9 minimal medium were supplemented with 4 μM TPEN, 200 μM metals (ZnSO_4_, FeCl_3_, CoCl_2_, or NiSO_4_), 25 μM EDTA, and/or 1 μM genistein as indicated in the text. Cultures were harvested 12 h later, and RNA was extracted using an RNAprep Pure Cell/Bacteria kit (Tiangen). Strand-specific RNA sequencing was carried out by Novogene (Beijing, China) with next-generation sequencing. For RNA-seq data, Bowtie2 was used to map clean reads in fastq files to the CCBAU45436 reference genome (default parameters) (73). The number of unique mapped reads for each protein-coding gene was extracted from sorted bam files using HTseq-count (74), and differentially expressed genes (Log2R > 1, false-discovery rate [FDR] < 0.001) were identified by DESeq2 (75). Summary statistics for the clean reads data and mapping results are shown in Table S1A.

Gene-specific primers used in qRT-PCR are listed in Table S2. cDNA was synthesized by using PrimeScript RT reagent kit with gDNA Eraser (TaKaRa). qRT-PCR was performed by using GoTaq qPCR Master Mix (Promega) and an ABI QuantStudio^T^ 6 Flex System real-time PCR system. Transcription levels were normalized to the expression of the internal control gene 16S rRNA, measured in the same samples. Three independent biological replicates were performed.

To determine the cotranscription of *znuB*, *znuC*, and *zur*, reverse transcription-PCR was conducted. Total RNA was extracted from the bacterial culture grown overnight at 28°C in modified M9 minimal medium. The RNA samples were first treated with gDNA Eraser (TaKaRa) to remove any contaminating genomic DNA, and then cDNA synthesis was performed using the PrimeScript RT reagent kit (TaKaRa). Gene-specific primers for the gene junctions were used for separate PCRs using the 2×High-GC PCR StarMix (GenStar). Three independent experiments were performed.

The translational fusion plasmid pGD926 with the *lacZ* reporter gene (76) was used to construct *P_znuA_-lacZ* in the backgrounds of CCBAU45436 and its *zur* mutant, using the procedure described earlier (59). To determine the effect of different zinc concentrations on *znuA* expression, cells from an overnight culture of WT/*P_znuA_-lacZ* and *zur*/*P_znuA_-lacZ* were washed once in M9 minimal medium and inoculated in 30 ml M9 minimal medium, supplemented with different ZnSO_4_ concentrations, to and OD_600_ of 0.05. β-Galactosidase activity was measured 16 h later as described previously (58).

### Determination of rhizobial growth and metal content.

Rhizobial cells were grown overnight in M9 minimal medium. The overnight cultures were washed once and resuspended to an OD_600_ of 0.05 in M9 minimal medium with or without 100 μM ZnSO_4_. Growth of cells was monitored by measuring optical density at 600 nm on the Bioscreen C system (Oy Growth Curves Ab Ltd., Finland). The overnight cultures were also used to inoculate M9 minimal medium containing 10 μM ZnSO_4_ or CoCl_2_ with an initial OD_600_ of 0.05. Strains were cultured for 48 h and were then pelleted, washed twice with 20 mM Tris-HCl (pH 7.4)–4 mM EDTA followed by one wash using 20 mM Tris-HCl (pH 7.4), and dried at 100°C to a constant weight. The total intracellular zinc or cobalt content was measured by inductively coupled plasma mass spectrometry (ICP-MS) (Agilent 7700x; Agilent, USA).

### Purification of *S. fredii* Zur, Western blotting, and electrophoretic mobility shift assay.

The PCR product of *zur* amplified using primers zur-ORF-F/zur-ORF-R was digested with BamHI and HindIII and ligated into expression vector pET-28a(+) to generate pET28a-Zur, which was then transformed into E. coli BL21(DE3). The expression of N-terminal His_6_-tagged Zur in E. coli cells grown in LB medium was induced with 1 mM (final concentration) isopropyl-β-d-thiogalactopyranoside (IPTG; Coolaber) for 4 h at 37°C. Cells were then harvested, washed, resuspended using lysis buffer (50 mM NaH_2_PO_4_, 300 mM NaCl) with protease inhibitor cocktail (Coolaber), and sonicated on ice. Cell extracts were loaded onto Nickel-IDA agarose beads (GenStar), and Zur protein was purified in accordance with the manufacturer’s instructions. The concentration of Zur protein was determined by the Bradford method using Quick Start Bradford Dye reagent (Bio-Rad).

For EMSA, CCBAU45436_3×Myc cells were cultured in the same way as bacteria used for qRT-PCR. Cell extracts were electrophoresed on 15% SDS-PAGE gels. Monoclonal mouse antibody against 3×Myc epitope (SC-40; Santa Cruz Biotechnology) and the horseradish peroxidase (HRP)-conjugated goat anti-mouse immunoglobulin G (IgG) secondary antibody (SC-2005; Santa Cruz Biotechnology) were used at 1:200 and 1:10,000 dilution ratios, respectively. Signals of the protein on X-ray film were recorded by chemiluminescence detection.

Binding reactions were performed with approximately 50 fmol of biotin-labeled probes and 2.64 μmol of purified Zur in 15 μl of the reaction buffer (10 mM Tris [pH 7.5], 50 mM KCl, 1 mM dithiothreitol [DTT], 1 μg of poly(dI-dC), and 200 μM ZnSO_4_, with or without 50 μM TPEN, followed by incubation at 25°C for 20 min. The binding mixture was subjected to electrophoresis at 4°C on 5% (wt/vol) native polyacrylamide gels at 150 V in TB (44.5 mM Tris, 44.5 mM boric acid) buffer for 1 h. After electrophoresis, DNA in the gels was transferred to a nylon membrane (Roche). Signals from biotin-labeled DNAs were detected by chemiluminescence through the imager apparatus (Alpha Flurechemical).

### Plant assays, cytological observation, and root surface colonization assay.

Seeds were surface sterilized using NaClO solution and allowed to germinate as described earlier (24, 77). Seedlings were inoculated using 1 ml of culture with an OD_600_ equivalent to 0.25 and grown in vermiculite moistened with low-N nutrient solution without zinc at 24°C under a day/night regimen of 12 h/12 h (57). When required, ZnSO_4_ or CoCl_2_ was added to the desired concentration. Forty days postinoculation (dpi), leaf chlorophyll content and shoot dry weight were determined using the procedure described earlier (24, 77). Nodules were harvested and fixed with 2.5% (vol/vol) glutaraldehyde in 0.05 M cacodylate buffer when necessary (78). For electron microscopy, ultrathin sections of fixed nodules were prepared and observed in a JEM-1230 transmission electron microscope (TEM) using procedures described previously (79). For light microscopy, root nodule sections were stained with 0.01% toluidine blue in 0.1 M phosphate buffer at pH 7.2 as previously described (80) and visualized under an Olympus BX53F light microscope.

For the colonization experiment, germinated soybean seeds were transferred to a sterile plastic petri dish (diameter, 9 cm) with low-N nutrient solution medium free of zinc, and inoculated with CCBAU45436 and its derivatives (1 ml culture with an OD_600_ equivalent to 1 in 0.85% NaCl solution for every plant). Plants were cultivated in a growth chamber at 24°C for 7 days under a day/night regimen of 12 h/12 h. Roots on plates containing bacteria were then sampled at 7 dpi, pooled, weighed, washed 7 times, and then suspended in 200 ml 0.85% NaCl solution. After exposure to 6 cycles of 30-s ultrasound treatment and agitation on a vortex for 2 min, the suspension was diluted and plated on TY plates with antibiotics. Colonies were counted 5 days after plating.

## References

[1] Andreini C, Banci L, Bertini I, Rosato A 2006 Zinc through the three domains of life. J Proteome Res 5:3173–3178. doi:10.1021/pr0603699. 17081069

[2] Hantke K 2001 Bacterial zinc transporters and regulators. Biometals 14:239–249. doi:10.1023/a:1012984713391. 11831459

[3] Hantke K 2005 Bacterial zinc uptake and regulators. Curr Opin Microbiol 8:196–202. doi:10.1016/j.mib.2005.02.001. 15802252

[4] Nies DH 2003 Efflux-mediated heavy metal resistance in prokaryotes. FEMS Microbiol Rev 27:313–339. doi:10.1016/S0168-6445(03)00048-2. 12829273

[5] Schmidt C, Schwarzenberger C, Große C, Nies DH 2014 FurC regulates expression of *zupT* for the central zinc importer ZupT of *Cupriavidus metallidurans*. J Bacteriol 196:3461–3471. doi:10.1128/JB.01713-14. 25049092 PMC4187674

[6] Chaoprasid P, Dokpikul T, Johnrod J, Sirirakphaisarn S, Nookabkaew S, Sukchawalit R, Mongkolsuk S 2016 *Agrobacterium tumefaciens* Zur regulates the high-affinity zinc uptake system TroCBA and the putative metal chaperone YciC, along with ZinT and ZnuABC, for survival under zinc-limiting conditions. Appl Environ Microbiol 82:3503–3514. doi:10.1128/AEM.00299-16. 27060116 PMC4959167

[7] Garrido ME, Bosch M, Medina R, Llagostera M, Pérez de Rozas AM, Badiola I, Barbé J 2003 The high-affinity zinc-uptake system *znuACB* is under control of the iron-uptake regulator (*fur*) gene in the animal pathogen *Pasteurella multocida*. FEMS Microbiol Lett 221:31–37. doi:10.1016/S0378-1097(03)00131-9. 12694907

[8] Sabri M, Houle S, Dozois CM 2009 Roles of the extraintestinal pathogenic *Escherichia coli* ZnuACB and ZupT zinc transporters during urinary tract infection. Infect Immun 77:1155–1164. doi:10.1128/IAI.01082-08. 19103764 PMC2643633

[9] Cerasi M, Liu JZ, Ammendola S, Poe AJ, Petrarca P, Pesciaroli M, Pasquali P, Raffatellu M, Battistoni A 2014 The ZupT transporter plays an important role in zinc homeostasis and contributes to *Salmonella enterica* virulence. Metallomics 6:845–853. doi:10.1039/c3mt00352c. 24430377 PMC3969385

[10] Gaballa A, Helmann JD 1998 Identification of a zinc-specific metalloregulatory protein, Zur, controlling zinc transport operons in *Bacillus subtilis*. J Bacteriol 180:5815–5821. doi:10.1128/JB.180.22.5815-5821.1998. 9811636 PMC107652

[11] Patzer SI, Hantke K 2000 The zinc-responsive regulator Zur and its control of the *znu* gene cluster encoding the ZnuABC zinc uptake system in *Escherichia coli*. J Biol Chem 275:24321–24332. doi:10.1074/jbc.M001775200. 10816566

[12] Shin JH, Oh SY, Kim SJ, Roe JH 2007 The zinc-responsive regulator Zur controls a zinc uptake system and some ribosomal proteins in *Streptomyces coelicolor* A3(2). J Bacteriol 189:4070–4077. doi:10.1128/JB.01851-06. 17416659 PMC1913400

[13] Li Y, Qiu Y, Gao H, Guo Z, Han Y, Song Y, Du Z, Wang X, Zhou D, Yang R 2009 Characterization of Zur-dependent genes and direct Zur targets in *Yersinia pestis*. BMC Microbiol 9:128. doi:10.1186/1471-2180-9-128. 19552825 PMC2706843

[14] Dahiya I, Stevenson RMW 2010 The ZnuABC operon is important for *Yersinia ruckeri* infections of rainbow trout, *Oncorhynchus mykiss* (Walbaum). J Fish Dis 33:331–340. doi:10.1111/j.1365-2761.2009.01125.x. 20070462

[15] Bhubhanil S, Sittipo P, Chaoprasid P, Nookabkaew S, Sukchawalit R, Mongkolsuk S 2014 Control of zinc homeostasis in *Agrobacterium tumefaciens* via *zur* and the zinc uptake genes *znuABC* and *zinT*. Microbiology 160:2452–2463. doi:10.1099/mic.0.082446-0. 25227896

[16] Ilari A, Alaleona F, Tria G, Petrarca P, Battistoni A, Zamparelli C, Verzili D, Falconi M, Chiancone E 2014 The *Salmonella enterica* ZinT structure, zinc affinity and interaction with the high-affinity uptake protein ZnuA provide insight into the management of periplasmic zinc. Biochim Biophys Acta 1840:535–544. doi:10.1016/j.bbagen.2013.10.010. 24128931

[17] Gabbianelli R, Scotti R, Ammendola S, Petrarca P, Nicolini L, Battistoni A 2011 Role of ZnuABC and ZinT in *Escherichia coli* O157:H7 zinc acquisition and interaction with epithelial cells. BMC Microbiol 11:36. doi:10.1186/1471-2180-11-36. 21338480 PMC3053223

[18] Hood MI, Skaar EP 2012 Nutritional immunity: transition metals at the pathogen-host interface. Nat Rev Microbiol 10:525–537. doi:10.1038/nrmicro2836. 22796883 PMC3875331

[19] Murphy TF, Brauer AL, Kirkham C, Johnson A, Koszelak-Rosenblum M, Malkowski MG 2013 Role of the zinc uptake ABC transporter of *Moraxella catarrhalis* in persistence in the respiratory tract. Infect Immun 81:3406–3413. doi:10.1128/IAI.00589-13. 23817618 PMC3754222

[20] Desrosiers DC, Bearden SW, Mier I, Abney J, Paulley JT, Fetherston JD, Salazar JC, Radolf JD, Perry RD 2010 Znu is the predominant zinc importer in *Yersinia pestis* during *in vitro* growth but is not essential for virulence. Infect Immun 78:5163–5177. doi:10.1128/IAI.00732-10. 20855510 PMC2981304

[21] Petrarca P, Ammendola S, Pasquali P, Battistoni A 2010 The Zur-regulated ZinT protein is an auxiliary component of the high-affinity ZnuABC zinc transporter that facilitates metal recruitment during severe zinc shortage. J Bacteriol 192:1553–1564. doi:10.1128/JB.01310-09. 20097857 PMC2832539

[22] Remigi P, Zhu J, Young JPW, Masson-Boivin C 2016 Symbiosis within symbiosis: evolving nitrogen-fixing legume symbionts. Trends Microbiol 24:63–75. doi:10.1016/j.tim.2015.10.007. 26612499

[23] Capela D, Marchetti M, Clérissi C, Perrier A, Guetta D, Gris C, Valls M, Jauneau A, Cruveiller S, Rocha EPC, Masson-Boivin C 2017 Recruitment of a lineage-specific virulence regulatory pathway promotes intracellular infection by a plant pathogen experimentally evolved into a legume symbiont. Mol Biol Evol 34:2503–2521. doi:10.1093/molbev/msx165. 28535261

[24] Liu LX, Li QQ, Zhang YZ, Hu Y, Jiao J, Guo HJ, Zhang XX, Zhang B, Chen WX, Tian CF 2017 The nitrate-reduction gene cluster components exert lineage-dependent contributions to optimization of *Sinorhizobium* symbiosis with soybeans. Environ Microbiol 19:4926–4938. doi:10.1111/1462-2920.13948. 28967174

[25] Zhao R, Liu LX, Zhang YZ, Jiao J, Cui WJ, Zhang B, Wang XL, Li ML, Chen Y, Xiong ZQ, Chen WX, Tian CF 2018 Adaptive evolution of rhizobial symbiotic compatibility mediated by co-evolved insertion sequences. ISME J 12:101–111. doi:10.1038/ismej.2017.136. 28800133 PMC5738999

[26] Jiao J, Ni M, Zhang B, Zhang Z, Young JPW, Chan T-F, Chen WX, Lam H-M, Tian CF 2018 Coordinated regulation of core and accessory genes in the multipartite genome of *Sinorhizobium fredii*. PLoS Genet 14:e1007428. doi:10.1371/journal.pgen.1007428. 29795552 PMC5991415

[27] Jiao J, Wu LJ, Zhang B, Hu Y, Li Y, Zhang XX, Guo HJ, Liu LX, Chen WX, Zhang Z, Tian CF 2016 MucR is required for transcriptional activation of conserved ion transporters to support nitrogen fixation of *Sinorhizobium fredii* in soybean nodules. Mol Plant Microbe Interact 29:352–361. doi:10.1094/MPMI-01-16-0019-R. 26883490

[28] Moreau S, Thomson RM, Kaiser BN, Trevaskis B, Guerinot ML, Udvardi MK, Puppo A, Day DA 2002 GmZIP1 encodes a symbiosis-specific zinc transporter in soybean. J Biol Chem 277:4738–4746. doi:10.1074/jbc.M106754200. 11706025

[29] Abreu I, Saéz Á, Castro-Rodríguez R, Escudero V, Rodríguez-Haas B, Senovilla M, Larue C, Grolimund D, Tejada-Jiménez M, Imperial J, González-Guerrero M 2017 *Medicago truncatula* Zinc-iron permease 6 provides zinc to rhizobia-infected nodule cells. Plant Cell Environ 40:2706–2719. doi:10.1111/pce.13035. 28732146

[30] Clarke VC, Loughlin PC, Day D. a, Smith PMC 2014 Transport processes of the legume symbiosome membrane. Front Plant Sci 5:699. doi:10.3389/fpls.2014.00699. 25566274 PMC4266029

[31] Okazaki S, Kaneko T, Sato S, Saeki K 2013 Hijacking of leguminous nodulation signaling by the rhizobial type III secretion system. Proc Natl Acad Sci U S A 110:17131–17136. doi:10.1073/pnas.1302360110. 24082124 PMC3801068

[32] Yasuda M, Miwa H, Masuda S, Takebayashi Y, Sakakibara H, Okazaki S 2016 Effector-triggered immunity determines host genotype-specific incompatibility in legume-rhizobium symbiosis. Plant Cell Physiol 57:1791–1800. doi:10.1093/pcp/pcw104. 27373538

[33] Sugawara M, Takahashi S, Umehara Y, Iwano H, Tsurumaru H, Odake H, Suzuki Y, Kondo H, Konno Y, Yamakawa T, Sato S, Mitsui H, Minamisawa K 2018 Variation in bradyrhizobial NopP effector determines symbiotic incompatibility with Rj2-soybeans via effector-triggered immunity. Nat Commun 9:3139. doi:10.1038/s41467-018-05663-x. 30087346 PMC6081438

[34] Yang S, Tang F, Gao M, Krishnan HB, Zhu H 2010 R gene-controlled host specificity in the legume-rhizobia symbiosis. Proc Natl Acad Sci U S A 107:18735–18740. doi:10.1073/pnas.1011957107. 20937853 PMC2973005

[35] Rehman HM, Cheung W-L, Wong K-S, Xie M, Luk C-Y, Wong F-L, Li M-W, Tsai S-N, To W-T, Chan L-Y, Lam H-M 2019 High-throughput mass spectrometric analysis of the whole proteome and secretome from *Sinorhizobium fredii* strains CCBAU25509 and CCBAU45436. Front Microbiol 10:2569. doi:10.3389/fmicb.2019.02569. 31798547 PMC6865838

[36] Ausmees N, Kobayashi H, Deakin WJ, Marie C, Krishnan HB, Broughton WJ, Perret X 2004 Characterization of NopP, a type III secreted effector of *Rhizobium* sp. strain NGR234. J Bacteriol 186:4774–4780. doi:10.1128/JB.186.14.4774-4780.2004. 15231809 PMC438593

[37] Skorpil P, Saad MM, Boukli NM, Kobayashi H, Ares-Orpel F, Broughton WJ, Deakin WJ 2005 NopP, a phosphorylated effector of *Rhizobium* sp strain NGR234, is a major determinant of nodulation of the tropical legumes *Flemingia congesta* and *Tephrosia vogelii*. Mol Microbiol 57:1304–1317. doi:10.1111/j.1365-2958.2005.04768.x. 16102002

[38] Kobayashi H, Naciri-Graven Y, Broughton WJ, Perret X, Graven YN 2004 Flavonoids induce temporal shifts in gene-expression of nod-box controlled loci in *Rhizobium* sp. NGR234. Mol Microbiol 51:335–347. doi:10.1046/j.1365-2958.2003.03841.x. 14756776

[39] Wassem R, Kobayashi H, Kambara K, Le Quéré A, Walker GC, Broughton WJ, Deakin WJ 2008 TtsI regulates symbiotic genes in *Rhizobium* species NGR234 by binding to *tts* boxes. Mol Microbiol 68:736–748. doi:10.1111/j.1365-2958.2008.06187.x. 18363648 PMC2770584

[40] Oldroyd GED 2013 Speak, friend, and enter: signalling systems that promote beneficial symbiotic associations in plants. Nat Rev Microbiol 11:252–263. doi:10.1038/nrmicro2990. 23493145

[41] Cai J, Zhang L-Y, Liu W, Tian Y, Xiong J-S, Wang Y-H, Li R-J, Li H-M, Wen J, Mysore KS, Boller T, Xie Z-P, Staehelin C 2018 Role of the nod factor hydrolase MtNFH1 in regulating nod factor levels during rhizobial infection and in mature nodules of *Medicago truncatula*. Plant Cell 30:397–414. doi:10.1105/tpc.17.00420. 29367305 PMC5868697

[42] Machado D, Pueppke SG, Vinardel JM, Ruiz-Sainz JE, Krishnan HB 1998 Expression of *nodD1* and *nodD2* in *Sinorhizobium fredii*, a nitrogen-fixing symbiont of soybean and other legumes. Mol Plant Microbe Interact 11:375–382. doi:10.1094/MPMI.1998.11.5.375.

[43] Garcia M, Dunlap J, Loh J, Stacey G 1996 Phenotypic characterization and regulation of the *nolA* gene of *Bradyrhizobium japonicum*. Mol Plant Microbe Interact 9:625–635. doi:10.1094/mpmi-9-0625. 8810078

[44] Fellay R, Hanin M, Montorzi G, Frey J, Freiberg C, Golinowski W, Staehelin C, Broughton WJ, Jabbouri S 1998 *nodD2* of *Rhizobium* sp. NGR234 is involved in the repression of the *nodABC* operon. Mol Microbiol 27:1039–1050. doi:10.1046/j.1365-2958.1998.00761.x. 9535093

[45] Vinardell JM, Ollero FJ, Hidalgo A, López-Baena FJ, Medina C, Ivanov-Vangelov K, Parada M, Madinabeitia N, Espuny M. d R, Bellogín RA, Camacho M, Rodríguez-Navarro D-N, Soria-Díaz ME, Gil-Serrano AM, Ruiz-Sainz JE 2004 NoIR regulates diverse symbiotic signals of *Sinorhizobium fredii* HH103. Mol Plant Microbe Interact 17:676–685. doi:10.1094/MPMI.2004.17.6.676. 15195950

[46] Blindauer CA 2015 Advances in the molecular understanding of biological zinc transport. Chem Commun (Camb) 51:4544–4563. doi:10.1039/c4cc10174j. 25627157

[47] Osman D, Foster AW, Chen J, Svedaite K, Steed JW, Lurie-Luke E, Huggins TG, Robinson NJ 2017 Fine control of metal concentrations is necessary for cells to discern zinc from cobalt. Nat Commun 8:1884. doi:10.1038/s41467-017-02085-z. 29192165 PMC5709419

[48] Wang D, Wang YC, Wu LJ, Liu JX, Zhang P, Jiao J, Yan H, Liu T, Tian CF, Chen WX 2016 Construction and pilot screening of a signature-tagged mutant library of *Sinorhizobium fredii*. Arch Microbiol 198:91–99. doi:10.1007/s00203-015-1161-9. 26472206

[49] Pawlik MC, Hubert K, Joseph B, Claus H, Schoen C, Vogel U 2012 The zinc-responsive regulon of *Neisseria meningitidis* comprises 17 genes under control of a Zur element. J Bacteriol 194:6594–6606. doi:10.1128/JB.01091-12. 23043002 PMC3497534

[50] Choi S-H, Lee K-L, Shin J-H, Cho Y-B, Cha S-S, Roe J-H 2017 Zinc-dependent regulation of zinc import and export genes by Zur. Nat Commun 8:15812. doi:10.1038/ncomms15812. 28598435 PMC5472717

[51] Andreini C, Bertini I, Rosato A 2009 Metalloproteomes: a bioinformatic approach. Acc Chem Res 42:1471–1479. doi:10.1021/ar900015x. 19697929

[52] Pueppke SG, Bolanos-Vasquez MC, Werner D, Bec-Ferte MP, Prome JC, Krishnan HB 1998 Release of flavonoids by the soybean cultivars McCall and Peking and their perception as signals by the nitrogen-fixing symbiont *Sinorhizobium fredii*. Plant Physiol 117:599–608. doi:10.1104/pp.117.2.599. 9625713 PMC34980

[53] Baglivo I, Russo L, Esposito S, Malgieri G, Renda M, Salluzzo A, Di Blasio B, Isernia C, Fattorusso R, Pedone PV 2009 The structural role of the zinc ion can be dispensable in prokaryotic zinc-finger domains. Proc Natl Acad Sci U S A 106:6933–6938. doi:10.1073/pnas.0810003106. 19369210 PMC2678482

[54] Tian CF, Zhou YJ, Zhang YM, Li QQ, Zhang YZ, Li DF, Wang S, Wang J, Gilbert LB, Li YR, Chen WX 2012 Comparative genomics of rhizobia nodulating soybean suggests extensive recruitment of lineage-specific genes in adaptations. Proc Natl Acad Sci U S A 109:8629–8634. doi:10.1073/pnas.1120436109. 22586130 PMC3365164

[55] Li QQ, Wang ET, Zhang YZ, Zhang YM, Tian CF, Sui XH, Chen WF, Chen WX 2011 Diversity and biogeography of rhizobia isolated from root nodules of *Glycine max* grown in Hebei province, China. Microb Ecol 61:917–931. doi:10.1007/s00248-011-9820-0. 21340735

[56] Zhang YM, Li Y, Chen WF, Wang ET, Tian CF, Li QQ, Zhang YZ, Sui XH, Chen WX 2011 Biodiversity and biogeography of rhizobia associated with soybean plants grown in the North China Plain. Appl Environ Microbiol 77:6331–6342. doi:10.1128/AEM.00542-11. 21784912 PMC3187167

[57] Vincent JM 1970 A manual for the practical study of root nodule bacteria. Blackwell, Oxford, United Kingdom.

[58] Miller JH 1972 Experiments in molecular genetics. Cold Spring Harbor Laboratory, Cold Spring Harbor, NY.

[59] Hu Y, Jiao J, Liu LX, Sun YW, Chen WF, Sui XH, Chen WX, Tian CF 2018 Evidence for phosphate starvation of rhizobia without terminal differentiation in legume nodules. Mol Plant Microbe Interact 31:1060–1068. doi:10.1094/MPMI-02-18-0031-R. 29663866

[60] Marx CJ, Lidstrom ME 2002 Broad-host-range *cre-lox* system for antibiotic marker recycling in Gram-negative bacteria. Biotechniques 33:1062–1067. doi:10.2144/02335rr01. 12449384

[61] Ditta G, Stanfield S, Corbin D, Helinski DR 1980 Broad host range DNA cloning system for Gram-negative bacteria: construction of a gene bank of *Rhizobium meliloti*. Proc Natl Acad Sci U S A 77:7347–7351. doi:10.1073/pnas.77.12.7347. 7012838 PMC350500

[62] Oke V, Long SR 1999 Bacterial genes induced within the nodule during the *Rhizobium*-legume symbiosis. Mol Microbiol 32:837–849. doi:10.1046/j.1365-2958.1999.01402.x. 10361286

[63] Quandt J, Hynes MF 1993 Versatile suicide vectors which allow direct selection for gene replacement in Gram-negative bacteria. Gene 127:15–21. doi:10.1016/0378-1119(93)90611-6. 8486283

[64] Kovach ME, Elzer PH, Hill DS, Robertson GT, Farris MA, Roop RM, Peterson KM 1995 Four new derivatives of the broad-host-range cloning vector pBBR1MCS, carrying different antibiotic-resistance cassettes. Gene 166:175–176. doi:10.1016/0378-1119(95)00584-1. 8529885

[65] Novichkov PS, Kazakov AE, Ravcheev DA, Leyn SA, Kovaleva GY, Sutormin RA, Kazanov MD, Riehl W, Arkin AP, Dubchak I, Rodionov DA 2013 RegPrecise 3.0 - a resource for genome-scale exploration of transcriptional regulation in bacteria. BMC Genomics 14:745. doi:10.1186/1471-2164-14-745. 24175918 PMC3840689

[66] Hertz GZ, Hartzell GW, Stormo GD 1990 Identification of consensus patterns in unaligned DNA sequences known to be functionally related. Comput Appl Biosci 6:81–92. doi:10.1093/bioinformatics/6.2.81. 2193692

[67] Medina-Rivera A, Defrance M, Sand O, Herrmann C, Castro-Mondragon JA, Delerce J, Jaeger S, Blanchet C, Vincens P, Caron C, Staines DM, Contreras-Moreira B, Artufel M, Charbonnier-Khamvongsa L, Hernandez C, Thieffry D, Thomas-Chollier M, van Helden J 2015 RSAT 2015: Regulatory Sequence Analysis Tools. Nucleic Acids Res 43:W50–W56. doi:10.1093/nar/gkv362. 25904632 PMC4489296

[68] Crooks GE, Hon G, Chandonia JM, Brenner SE 2004 WebLogo: a sequence logo generator. Genome Res 14:1188–1190. doi:10.1101/gr.849004. 15173120 PMC419797

[69] Chenna R, Sugawara H, Koike T, Lopez R, Gibson TJ, Higgins DG, Thompson JD 2003 Multiple sequence alignment with the Clustal series of programs. Nucleic Acids Res 31:3497–3500. doi:10.1093/nar/gkg500. 12824352 PMC168907

[70] Kumar S, Stecher G, Tamura K 2016 MEGA7: Molecular Evolutionary Genetics Analysis version 7.0 for bigger datasets. Mol Biol Evol 33:1870–1874. doi:10.1093/molbev/msw054. 27004904 PMC8210823

[71] Biasini M, Bienert S, Waterhouse A, Arnold K, Studer G, Schmidt T, Kiefer F, Gallo Cassarino T, Bertoni M, Bordoli L, Schwede T 2014 SWISS-MODEL: modelling protein tertiary and quaternary structure using evolutionary information. Nucleic Acids Res 42:W252–W258. doi:10.1093/nar/gku340. 24782522 PMC4086089

[72] Castresana J 2000 Selection of conserved blocks from multiple alignments for their use in phylogenetic analysis. Mol Biol Evol 17:540–552. doi:10.1093/oxfordjournals.molbev.a026334. 10742046

[73] Langmead B, Salzberg SL 2012 Fast gapped-read alignment with Bowtie 2. Nat Methods 9:357–359. doi:10.1038/nmeth.1923. 22388286 PMC3322381

[74] Anders S, Pyl PT, Huber W 2015 HTSeq-a Python framework to work with high-throughput sequencing data. Bioinformatics 31:166–169. doi:10.1093/bioinformatics/btu638. 25260700 PMC4287950

[75] Love MI, Huber W, Anders S 2014 Moderated estimation of fold change and dispersion for RNA-seq data with DESeq2. Genome Biol 15:550. doi:10.1186/s13059-014-0550-8. 25516281 PMC4302049

[76] Ditta G, Schmidhauser T, Yakobson E, Lu P, Liang XW, Finlay DR, Guiney D, Helinski DR 1985 Plasmids related to the broad host range vector, pRK290, useful for gene cloning and for monitoring gene-expression. Plasmid 13:149–153. doi:10.1016/0147-619x(85)90068-x. 2987994

[77] Li YZ, Wang D, Feng XY, Jiao J, Chen WX, Tian CF 2016 Genetic analysis reveals the essential role of nitrogen phosphotransferase system components in *Sinorhizobium fredii* CCBAU 45436 symbioses with soybean and pigeonpea plants. Appl Environ Microbiol 82:1305–1315. doi:10.1128/AEM.03454-15. 26682851 PMC4751829

[78] Van de Velde W, Guerra JCP, De Keyser A, De Rycke R, Rombauts S, Maunoury N, Mergaert P, Kondorosi E, Holsters M, Goormachtig S 2006 Aging in legume symbiosis. A molecular view on nodule senescence in *Medicago truncatula*. Plant Physiol 141:711–720. doi:10.1104/pp.106.078691. 16648219 PMC1475454

[79] Li Y, Tian CF, Chen WF, Wang L, Sui XH, Chen WX 2013 High-resolution transcriptomic analyses of *Sinorhizobium* sp. NGR234 bacteroids in determinate nodules of *Vigna unguiculata* and indeterminate nodules of *Leucaena leucocephala*. PLoS One 8:e70531. doi:10.1371/journal.pone.0070531. 23936444 PMC3732241

[80] Ronson CW, Lyttleton P, Robertson JG 1981 C4-dicarboxylate transport mutants of *Rhizobium trifolii* form ineffective nodules on *Trifolium repens*. Proc Natl Acad Sci U S A 78:4284–4288. doi:10.1073/pnas.78.7.4284. 16593058 PMC319774

