# Supplementary material for: Modulation of Symbiotic Compatibility by Rhizobial Zinc Starvation Machinery
Source: mBio. 2020 Feb 18;11(1):e03193-19. doi: 10.1128/mBio.03193-19 (PMC7029138; doi:10.1128/mBio.03193-19)
Supplement: FIG S1 [file mBio.03193-19-sf001.pdf]

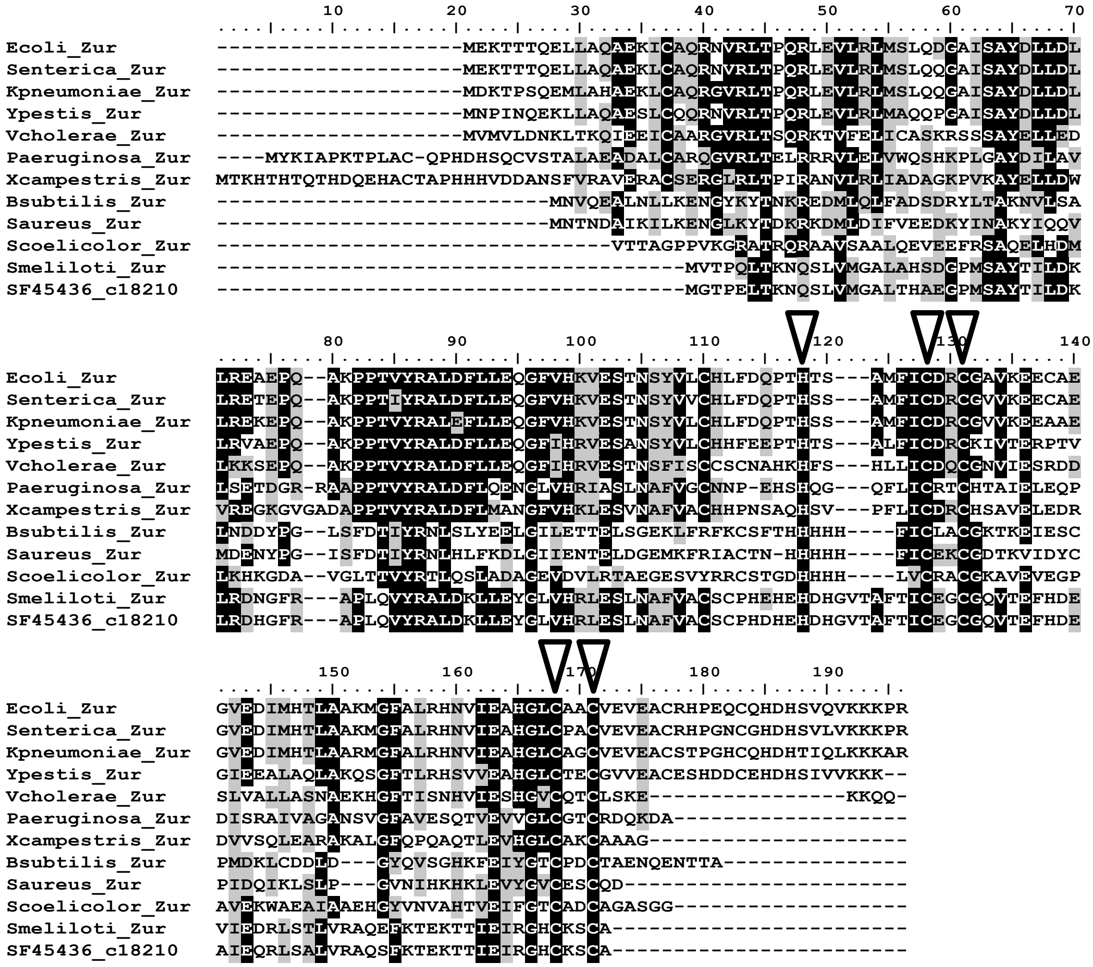

Fig. S1. Sequence alignment of c18210 with other bacterial Zur proteins. The protein sequence of SF45436_c18210 was aligned with Zur proteins from the following bacteria: *Escherichia coli, Salmonella enterica, Klebsiella pneumoniae, Yersinia pestis, Vibrio cholerae, Pseudomonas aeruginosa, Xanthomonas campestris, Bacillus subtilis, Staphylococcus aureus* using ClustalW. Five conserved residues (H77, C87, C90, C128, and C130) that are critical for the specific binding to zinc ions are indicated with triangles.
