# Supplementary material for: Modulation of Symbiotic Compatibility by Rhizobial Zinc Starvation Machinery
Source: mBio. 2020 Feb 18;11(1):e03193-19. doi: 10.1128/mBio.03193-19 (PMC7029138; doi:10.1128/mBio.03193-19)
Supplement: FIG S2 [file mBio.03193-19-sf002.pdf]

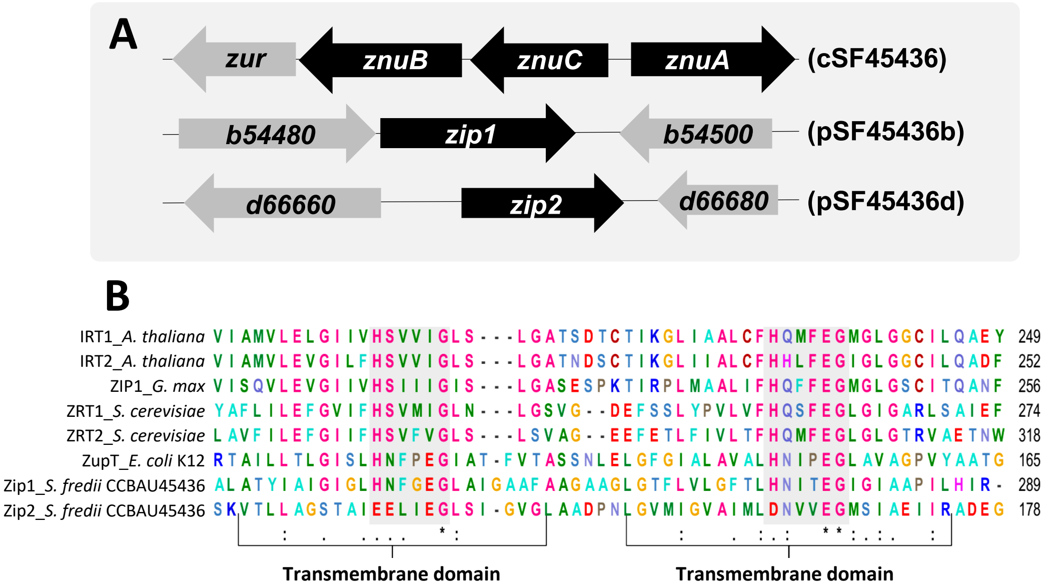

Fig. S2. Putative zinc uptake transporters encoded in the multipartite genome of *S. fredii* CCBAU45436. (**A**) Genes encoding putative transporters ZnuABC, Zip1, and Zip2 are shown in black. These genes are located on three of the five replicons of CCBAU45436: cSF45436 (chromosome), pSF45436b (chromid), and pSF45436d (an accessory plasmid). (**B**) Zip1 and Zip2 of CCBAU45436 harbor two characterized conserved regions (shaded) in two transmembrane domains of ZIP family proteins. The partial protein sequences of Zip1 (SF45436_b54490) and Zip2 (SF45436_d66670) were aligned with ZIP protein homologs from *Arabidopsis thaliana*, *Saccharomyces cerevisiae*, *Glycine max* and *Escherichia coli.*
