# Supplementary material for: Modulation of Symbiotic Compatibility by Rhizobial Zinc Starvation Machinery
Source: mBio. 2020 Feb 18;11(1):e03193-19. doi: 10.1128/mBio.03193-19 (PMC7029138; doi:10.1128/mBio.03193-19)
Supplement: FIG S3 [file mBio.03193-19-sf003.pdf]

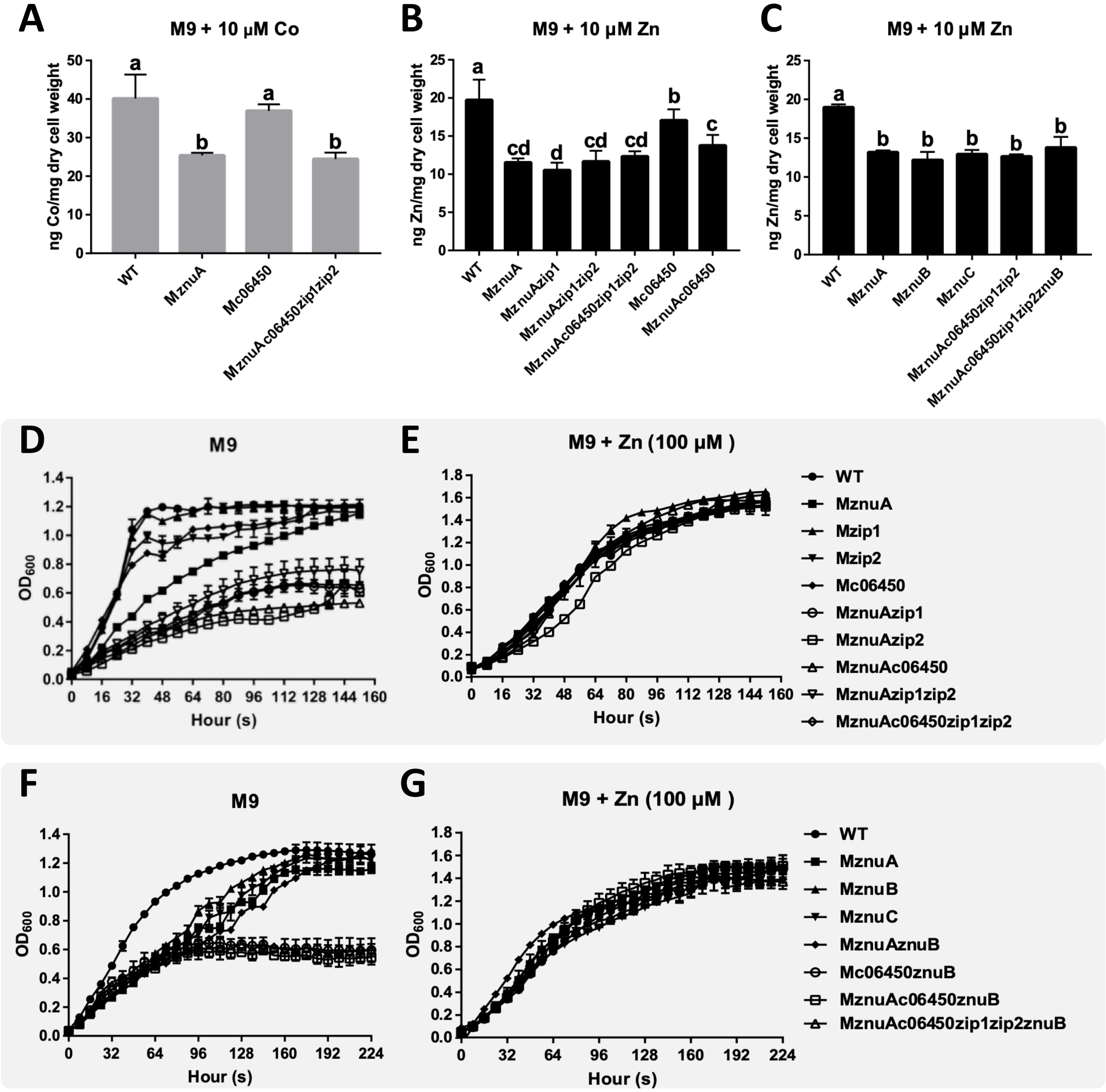

Fig. S3. Znu as the major zinc uptake system under zinc-deplete conditions. (**A**) Intracellular cobalt content of strains grown in M9 medium supplied with 10 μM $CoCl_2$. (**B-C**) Intracellular zinc content of strains grown in M9 medium supplied with 10 μM $ZnSO_4$. (**D-G**) Growth curves determined in M9 medium without (**C, F**) or with 100 μM $ZnSO_4$ (**D, G**). Values are mean ± SD of biological triplicates. Different letters indicate significant difference (Duncan test, alpha = 0.05).
