# Supplementary material for: Modulation of Symbiotic Compatibility by Rhizobial Zinc Starvation Machinery
Source: mBio. 2020 Feb 18;11(1):e03193-19. doi: 10.1128/mBio.03193-19 (PMC7029138; doi:10.1128/mBio.03193-19)
Supplement: FIG S4 [file mBio.03193-19-sf004.pdf]

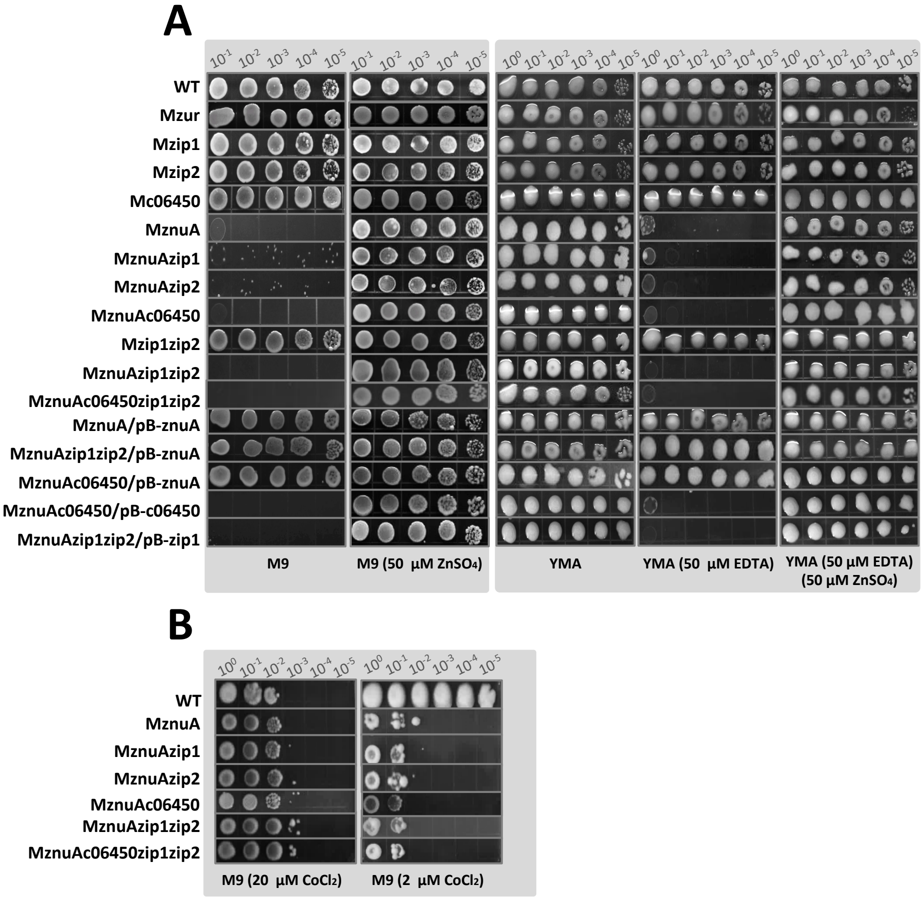

Fig. S4. Znu is essential for low zinc adaptation. (**A**) Cell suspension of OD_{600} = 1.0 was serially diluted and spotted onto plates with the M9 minimal medium with or without 50 µM $ZnSO_4$, or plates with the YMA medium supplemented with 50 µM EDTA, or 50 µM EDTA and 50 µM $ZnSO_4$. Plasmids pB-*znuA,* pB-*zip1* and pB-*c06450* were used in complementation experiments*.* (**B**) Growth defects of *znu* mutants on M9 plates can be partially restored by adding $CoCl_2$. Cell suspension of OD_{600} = 1.0 was serially diluted and spotted onto plates with the M9 medium supplemented with 20 µM or 2 µM $CoCl_2$.
