# Supplementary material for: Modulation of Symbiotic Compatibility by Rhizobial Zinc Starvation Machinery
Source: mBio. 2020 Feb 18;11(1):e03193-19. doi: 10.1128/mBio.03193-19 (PMC7029138; doi:10.1128/mBio.03193-19)
Supplement: FIG S5 [file mBio.03193-19-sf005.pdf]

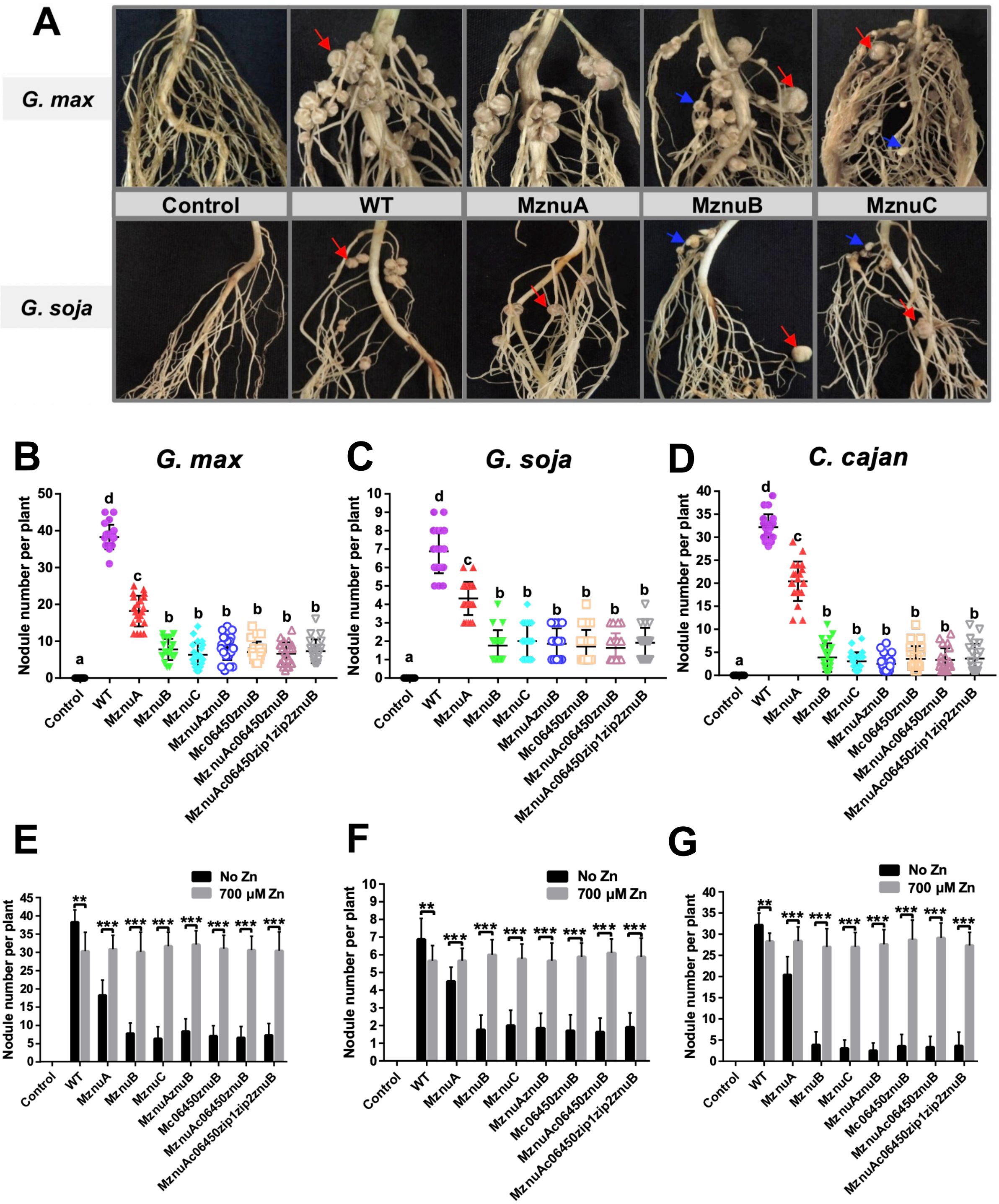

Fig. S5. Comparative analysis of nodulation phenotypes of mutants lacking different *znu* genes. (**A**) Representative photos showing infected nodules (red arrows) and bumps (blue arrows) observed on roots of *G. max* and *G. soja* inoculated with corresponding strains. (**B-D**) Number of infected nodules per plant of *G. max* (**B**), *G. soja* (**C**) and *C. cajan* (**D**). Means ± SD are based on 20-30 scored plants from three independent experiments. Different letters indicate significant difference (Duncan test, alpha = 0.05). (**E-G**) Number of infected nodules per plant of *G. max* (**E**), *G. soja* (**F**) and *C. cajan* (**G**) inoculated with corresponding strains under conditions with or without 700 μM $ZnSO_4$ in vermiculite moistened with low-N nutrient solution. Means ± SD are based on 20-30 scored plants from three independent experiments (*t* test; **, P < 0.01; ***, P < 0.001).
