# Supplementary material for: Modulation of Symbiotic Compatibility by Rhizobial Zinc Starvation Machinery
Source: mBio. 2020 Feb 18;11(1):e03193-19. doi: 10.1128/mBio.03193-19 (PMC7029138; doi:10.1128/mBio.03193-19)
Supplement: FIG S6 [file mBio.03193-19-sf006.pdf]

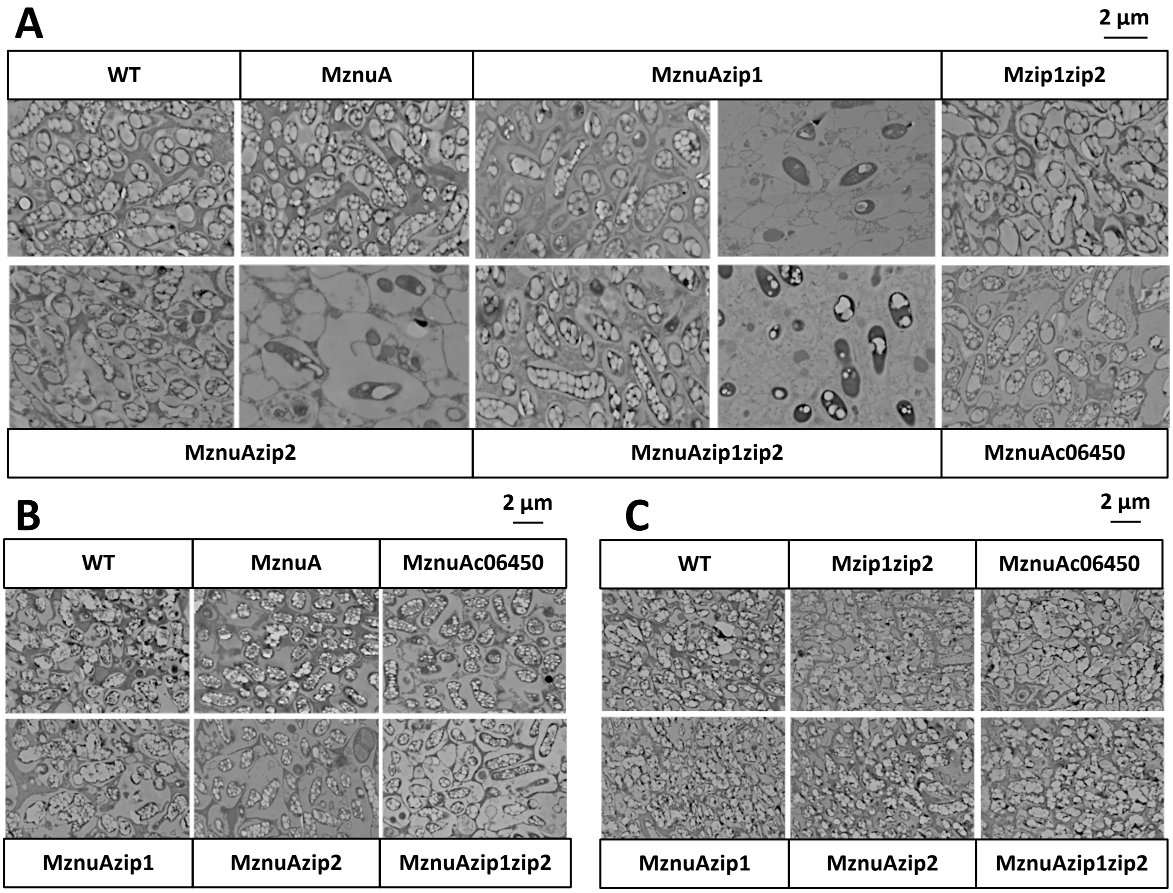

Fig. S6. Ultrathin sections of *G. max* (A), *G. soja* (B) and *C. cajan* (C) nodules. Pictures of ultrathin sections of 40 days post inoculation (dpi) nodules infected by CCBAU45436 and representative mutants were obtained by transmission electron microscopy. Scale bar indicates 2 µm.
