# Supplementary material for: Modulation of Symbiotic Compatibility by Rhizobial Zinc Starvation Machinery
Source: mBio. 2020 Feb 18;11(1):e03193-19. doi: 10.1128/mBio.03193-19 (PMC7029138; doi:10.1128/mBio.03193-19)
Supplement: FIG S7 [file mBio.03193-19-sf007.pdf]

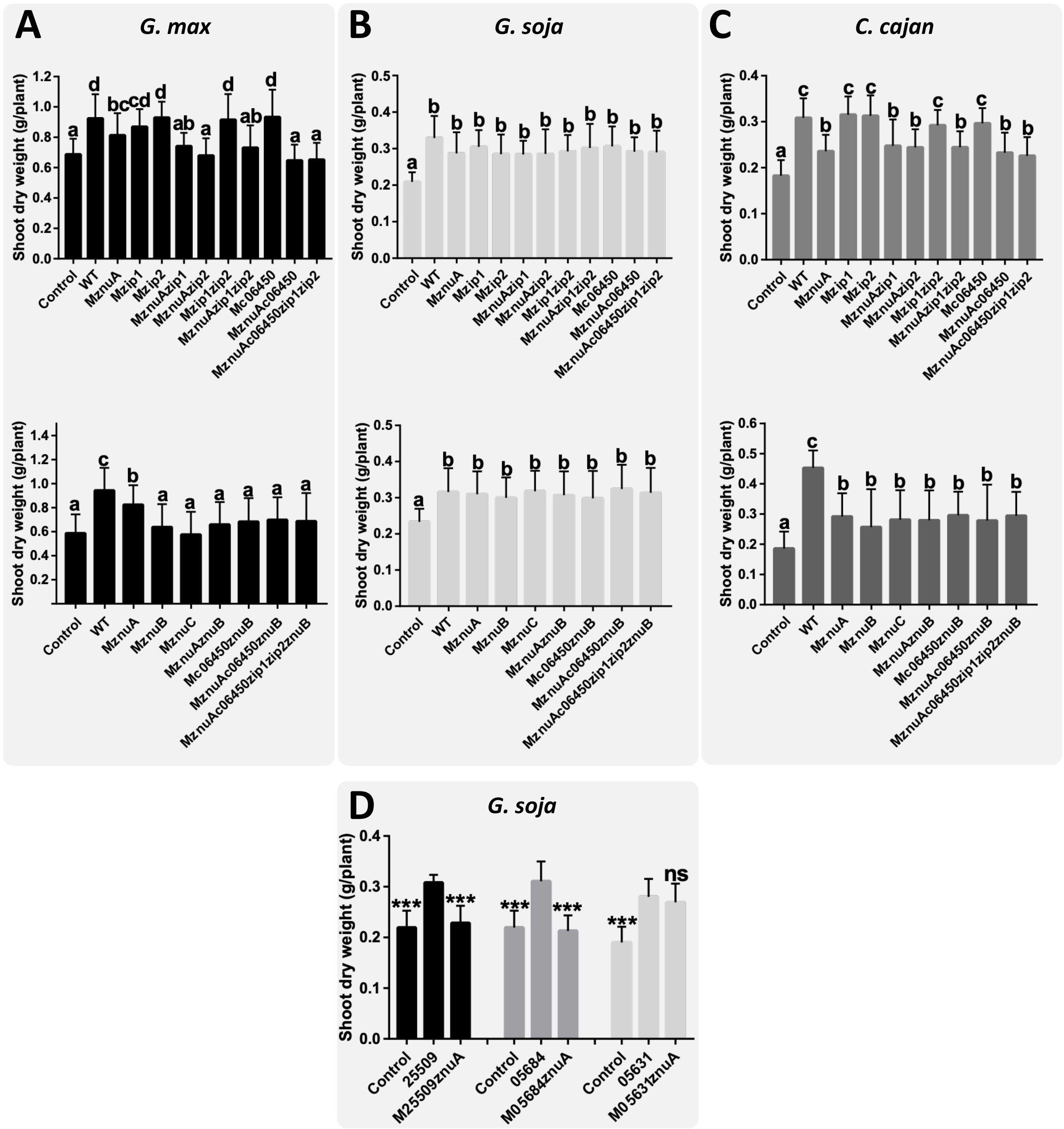

Fig. S7. Symbiotic performance of mutants lacking individual or multiple zinc transporter genes. (**A-C**) Shoot dry weight of *G. max* (**A**), *G. soja* (**B**) and *C. cajan* (**C**) plants inoculated with *S. fredii* CCBAU45436 and its derivatives lacking individual or multiple zinc transporter genes. (**D**) Shoot dry weight of *G. soja* plants inoculated with CCBAU25509/CCBAU05684/CCBAU05631 and their *znuA* mutants. Means ± SD are based on 20-30 scored plants from three independent experiments. Different letters indicate significant difference (Duncan test, alpha = 0.05). ***, P < 0.001 in *t* test of the mean comparison with that of wild-type *S. fredii* CCBAU25509, *S. sojae* CCBAU05684 or *Sinorhizobium* sp. CCBAU05631; ns, nonsignificant.
