# Supplementary material for: Modulation of Symbiotic Compatibility by Rhizobial Zinc Starvation Machinery
Source: mBio. 2020 Feb 18;11(1):e03193-19. doi: 10.1128/mBio.03193-19 (PMC7029138; doi:10.1128/mBio.03193-19)
Supplement: FIG S8 [file mBio.03193-19-sf008.pdf]

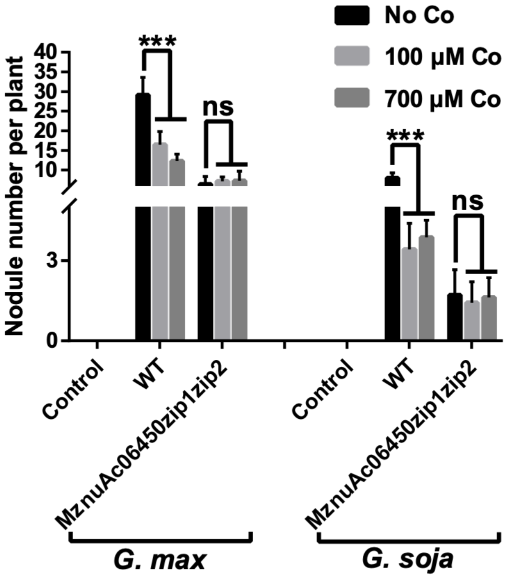

Fig. S8. Nodulation defects of mutants lacking *znu* cannot be recovered by replete cobalt. Nodule number per plant of *Glycine max* (**A**) and *Glycine soja* (**B**) were recorded under conditions with different concentrations of cobalt being supplied. Means ± SD are based on more than 20-30 scored plants. Significant effects of cobalt addition are indicated (*t* test; ns, $P > 0.05$; ***, $P < 0.001$). There were no nodules formed on *Cajanus cajan* plants inoculated with either WT or the mutant when cobalt was added.
